# Genome-wide identification, phylogenetic and expression pattern analysis of GATA gene family in *Cerasus humilis*

**DOI:** 10.3389/fpls.2025.1596930

**Published:** 2025-06-05

**Authors:** Meitong Pan, Chenzhuo Yue, Shan Jiang, Junbai Ma, Lengleng Ma, Lingyang Kong, Yang Ling, Wei Ma, Weichao Ren, Xiubo Liu

**Affiliations:** ^1^ College of Pharmacy, Heilongjiang University of Chinese Medicine, Harbin, China; ^2^ Functional Natural Products Research Center, Yichun Branch of Heilongjiang Academy of Forestry, Yichun, China; ^3^ College of Jiamusi, Heilongjiang University of Chinese Medicine, Jiamusi, China

**Keywords:** *Cerasus humilis*, GATA, gene expression, gene function, abiotic stress

## Abstract

**Introduction:**

The transcription factor GATA plays a pivotal role in plant growth, physical and metabolic functions, and responses to changes in the environment. After the completion of the *Cerasus humilis* genome, investigations into its GATA gene family were not pursued.

**Methods:**

Our research team identified the GATA gene’s bioinformatics techniques, analyzed its structural characteristics and evolutionary trajectory, and investigated its expression patterns among various subfamilies.

**Results:**

In the *C. humilis* genome,20 *ChGATAs* are divided into four unique subgroups distributed over 7 chromosomes. Collinearity analysis showed 7 pairs of segmental duplications and 2 pairs of tandem repeats. The duplication of these fragments is vital for the development of the ChGATA family. The evolutionary connection between *C. humilis* and the *Malus pumila* GATA gene family is more evident than with *Oryza sativa*. Moreover, the promoter region of the ChGATA gene family contained cis-acting elements linked to stress, hormones, and plant growth. The transcriptome heatmap showed that the expression of the *ChGATA* was specific under alkali stress, and *ChGATAs* in the same subpopulation would also show different expression patterns. qPCR analysis showed that most of the screened *ChGATAs* first decreased and then increased with time. In addition, the dual luciferase assay and protein interaction prediction showed that *ChWRKY29* could activate the expression of the differentially expressed gene *ChGATA14* in response to alkali stress, and ChGATA14 was at the core of the protein interaction network and had a strong interaction with ChGATA2 and ChGATA16.

**Discussion:**

This study laid a theoretical and scientific foundation for further studies on the biological function of *ChGATA*.

## 1 Introduction

Transcription factors (TF), often known as trans activators, are proteins capable of engaging with cis-acting components within a gene’s promoter. Transcriptional control in eukaryotes is frequently linked to cis-acting components, including enhancers, promoters, and silencers (Riechmann et al., 2000; Fan et al., 2021). TFs are crucial in the development of plants, hormone signaling routes, and disease management. There are several well-known families of transcription factors in plants, including WRKY, MYB, DREB, bZIP, and GATA. GATA transcription factor possesses the type IV zinc finger motifs, which are composed of one or two highly conserved zinc-finger domains (C-X_2_-CX_17_-_20_-C-X_2_-C; Type IVb) and DNA-binding domains (Lowry et al., 2000). The C-X_17–20_ domain of the type IV zinc finger structure differs among different eukaryotes. In fungi, most GATA transcription factors’ known type IV zinc finger structure is C-X_17_ (Teakle et al., 2002; Behringer and Schwechheimer, 2015). Typically, the GATA factors in animals contain two conserved zinc finger areas, C-X_2_-C-X_17_-C-X_2_-C. However, solely the C-terminal fingers play a role in DNA attachment, which is crucial for cellular division, growth, and development. Most plant GATA factors are limited to a single C-X_2_-C-X_18_-C-X_2_-C or CX_2_-C-X_20_-C-X_2_-C zinc finger domain. The *Arabidopsis thaliana* GATA family genes are generally divided into subfamilies I, II, III, and IV based on phylogenetic relationships, DNA domains, and intron-exon structures (Reyes et al., 2004; Yuan et al., 2018; Zhang et al., 2018, 2019, 2019).

A multitude of studies has identified a link between GATA TFs and phenomena such as plant development, flowering, chlorophyll generation, greening, and ageing. Furthermore, transcription factors linked to GNC (GATA21) and GNL (GATA22), facilitated by Phytochrome-interacting factors (PIF), are instrumental in regulating seed sprouting (Naito et al., 2007). Elevated levels of GNC and GNL may slow down the sprouting of *Arabidopsis thaliana* seeds, whereas deactivating GNC and GNL may enhance their germination, indicating a detrimental regulatory function of GNC and GNL in seed sprouting. The interaction of GNC/GNL with *SOC1*, a MADS-box transcription factor that inhibits Constans1 overexpression, affects the timing of flowering (Richter et al., 2013a; Zubo et al., 2018). Additionally, GNC and GNL markedly impede gibberellin signaling through regulation by DELLA and PIF regulators (Naito et al., 2007; Richter et al., 2010). GATA TFs are crucial in plants’ reactions to non-living stressors. As an example, under saline stress conditions, the *Oryza sativa OsGATA8* overexpression variants showed enhanced light efficiency and biomass relative to both standard and altered plants (Nutan et al., 2020). A significant decrease in the levels of *GATA44* and *GATA58* was observed in *Glycine max* seedlings under nitrogen scarcity (Zhang et al., 2015).

The little perennial deciduous shrub *Cerasus humilis* (*C. humilis*) is a member of the Rosaceae family. *C. humilis* fruit, rich in minerals advantageous for human health, has long been used in traditional Chinese medicine (Song et al., 2011; Yin et al., 2012). *C. humilis* is now recognized as a versatile fruit tree, offering extensive potential for various uses. Land is vital for the productivity and sustainable development of human societies. However, the escalation in population and significant greenhouse gas emissions have escalated global temperatures and climate change, exacerbating soil salinization and hindering plant growth and development (Mahajan and Tuteja, 2005; Fedoroff et al., 2010). Soil salinization is a major environmental factor that adversely affects agricultural output and the stability of food sources (Munns et al., 2008). As a reaction to shifts in the environment, plants have developed intricate communication routes, typically involving receptors, secondary signals, plant hormones, and signal transduction mechanisms (Liu, 2014). However, there are few studies on the mechanism of salt-alkali tolerance of *C. humilis*. Consequently, this research employed bioinformatics techniques to pinpoint GATA gene family members in *C. humilis*, examining their physical and chemical traits, gene architecture, phylogenetic links, promoter cis-acting components, and expression trends in alkali stress. In addition, the differentially expressed gene *ChGATA14* was screened based on the previous transcriptome sequencing results, and the transcriptional activation of *ChWRKY29* was determined by dual luciferase assay. This study provided the theoretical basis and technical support for genetic improvement and alkali-tolerant variety breeding of *C. humilis*, accelerated the cultivation of excellent varieties that adapt to different saline-alkali environments, and promoted the development of *C. humilis* industry in saline-alkali areas.

## 2 Materials and methods

### 2.1 Genome-wide identification of GATA gene family in *C. humilis*

*C. humilis* genome and gene annotation file downloaded from the national genome database (https://ngdc.cncb.ac.cn/databases) (Zhao et al., 2022). From the National Center for Biotechnology Information (https://www.ncbi.nlm.nih.gov), download *Arabidopsis thaliana*, *Malus pumila*, *Vitis vinifera*, and the *Oryza sativa* genome file. By employing the TBtools BLAST for analyzing homologous comparisons, 30 *Arabidopsis thaliana* GATA protein sequence members were chosen from the TAIR10 database to pinpoint the *C. humilis* GATA gene families. (score ≥100,e ≤1e−10) (Altschul et al., 1997). HM-MER3.0 was used to search for the unique proteins containing PF00032 protein structure, and the redundant sequences between HMMsearch results and BLASTP results were removed (Finn et al., 2011). Within the GATA gene family, preservation of only the sequence with the longest amino acid occurs when a single gene generates multiple distinct protein sequences. Following this, all protein sequences are gathered using the NCBI-CDD and SMART online tools, which artificially eliminate incorrect sequences. Finally, 20 members of the ChGATA gene family were obtained, and they were named according to their order of arrangement on the *C. humilis* genome chromosome. (**Table 1**). The ExPASy-ProtParam tool (http://web.expasy.org/protparam/) is employed to ascertain the isoelectric point (pI) and molecular weight (kDa) of the ChGATA protein. Subsequently, the WoLF PSORT online tool (https://wolfpsort.hgc.jp/) was utilized to forecast its subcellular positioning; the biotype is plant.

**Table 1:** List of the *ChGATAs* identified in the study.

Gene ID	Gene name	PI	MW	Subcellular location
*Chumilis00510.1*	*ChGATA1*	4.83	38401.46	nucleus
*Chumilis00511.1*	*ChGATA2*	5.61	31945.13	nucleus
*Chumilis01338.1*	*ChGATA3*	6.30	42288.63	nucleus
*Chumilis01670.1*	*ChGATA4*	9.08	37271.84	nucleus
*Chumilis04435.1*	*ChGATA5*	9.69	14912.09	nucleus
*Chumilis05700.1*	*ChGATA6*	9.63	20001.48	nucleus
*Chumilis06509.1*	*ChGATA7*	7.52	34515.57	nucleus
*Chumilis08509.1*	*ChGATA8*	9.50	19338.92	nucleus
*Chumilis10293.1*	*ChGATA9*	5.75	28115.91	nucleus
*Chumilis11028.1*	*ChGATA10*	6.01	37584.15	nucleus
*Chumilis14463.1*	*ChGATA11*	8.37	31533.64	chloroplast
*Chumilis14552.1*	*ChGATA12*	6.18	43512.55	chloroplast
*Chumilis15041.1*	*ChGATA13*	9.38	36454.03	nucleus
*Chumilis18605.1*	*ChGATA14*	6.05	31632.27	nucleus
*Chumilis19001.1*	*ChGATA15*	6.05	35334.07	nucleus
*Chumilis22132.2*	*ChGATA16*	5.39	31402.99	nucleus
*Chumilis22133.1*	*ChGATA17*	5.02	40285.85	nucleus
*Chumilis22257.1*	*ChGATA18*	5.49	35446.53	nucleus
*Chumilis24079.1*	*ChGATA19*	9.64	14285.56	nucleus
*Chumilis25938.1*	*ChGATA20*	6.53	60237.77	nucleus

### 2.2 Identifying genes in chromosomes and examining the tertiary structure of GATA family gene proteins in *C. humilis*

Utilizing the gene location visualization from the GTF/GFF feature of TBtools software, chromosome localization was executed, referencing the genome annotation file of *C. humilis*. The chromosome Gene Density was obtained using the Gene Density Profile to populate the chromosome. Employ the web-based utilities SWISS - MODEL to predict the tertiary structure of proteins using GATA protein sequences. The model with the GMQE value closest to 1 was used for modelling.

### 2.3 Aligning multiple sequences and conducting phylogenetic studies

Multiple amino acid sequence alignments were performed in MEGA11 using the default ClustalW parameters to eliminate sequences of different transcripts (Thompson et al., 2002). The neighborhood joining (NJ) method was used to construct the unrooted phylogenetic tree, and the parameter Settings included: The evolutionary distance was p-distance, which was based on the number of amino acid differences per site, the missing data method was Partial deletion, the cut off was 50%, the Bootstrap was set to 1000, and the other parameters were the default parameter (Tamura et al., 2021). The evolution tree is beautified by the online software Evolview.

### 2.4 Gene collinearity and evolutionary examination

The *ChGATA* location was derived from the *C. humilis* annotation GFF file, and the interspecific collinear relationship of *ChGATA* was plotted using Circos of TBtools. MCScanX facilitated the examination of *GATA* collinearity in *Arabidopsis thaliana*, *Malus pumila*, *Vitis vinifera*, *Oryza sativa*, and *C. humilis*. These findings were depicted using TBtools (v2.0697) in the Dual Synteny Plot (Jiang et al., 2023).

### 2.5 Investigating the genetic structure and patterns of proteins

The intron-exon configuration of the *ChGATA* in *C. humilis* was obtained from its genomic GFF annotation file. Utilizing the online MEME tool, we identified the GATA protein’s conserved motif in *C. humilis*. The optimized parameters were: locus distribution: 0 or 1 for each sequence; motif width: 6-50; Number of patterns:10; Default values for other parameters (Bailey et al., 2006). TBtools software was utilized to display the exon/intron configuration and preserved motifs of the *C. humilis* GATA protein (Chen et al., 2020).

### 2.6 Analysis of cis-acting element

The promoter region of each gene, situated at 1500 base pairs before the transcription initiation site, was isolated from the *C. humilis*’ genomic sequence using TBtools software (Chen et al., 2020). Subsequently, the PlantCARE software facilitated the examination of the cis-acting segments in the promoter area (Lescot et al., 2002). Finally, the results were visualized using the Simple BioSequence Viewer of TBtools software (Chen et al., 2020).

### 2.7 RNA extraction and quantitative RT-PCR analysis

Two-year-old healthy *C. humilis* seedlings (variety: Red Harpo 1) were used as experimental materials. The experimental materials were divided into control and alkali-treated groups for 4 and 8 days with three biological replicates per group. The control group was irrigated with distilled water, and the experimental group was irrigated with 200 mM sodium bicarbonate solution once a day. Leaves were collected after stress and then immediately frozen in liquid nitrogen and stored at minus 80°C. Control and treated 8-d seedlings were used for transcriptome, and three biological replicates were performed for each stage. Total RNA was isolated from these specimens utilizing FreeZol Reagent R711 in compliance with the prescribed manufacturer’s instructions. In essence, 50mg of the ground sample was mixed with 500 mL of FreeZol reagent for cracking. After centrifuging the lysate, the supernatant was then gathered. Subsequently, the dilution buffer was applied to the supernatant, and the mixture was precipitated using isopropyl alcohol. Post-centrifugation, the supernatant was disposed of, rinsed with 75% ethanol, and dissolved in double distilled water (ddH_2_O) without RNase. Evaluation of the isolated RNA samples’ integrity and concentration was conducted through 1% agarose gel electrophoresis and the use of an ultramicro nucleic acid concentration detector.

Primer 3 website (https://primer3.org/) was used for primer design (**Supplementary Table 1**). Standardized Actin primers are used as internal parameters (Livak and Schmittgen, 2001). Every test hole holds a reaction mixture of 20 μL at 95°C for one minute, succeeded by 40 repetitions at 95°C for ten seconds and 60°C for thirty seconds, forming a thermal cycle graph. After the last PCR cycle, the temperature was raised from 55°C to 99°C at a rate of 0.5°C/s to generate the melting curve of the sample. Gene expression was measured by the 2^-△△Ct^ method (Lynch and Conery, 2000). Each reaction was repeated three times, and the results were expressed as the mean of three independent biological replicates.

### 2.8 Analysis of gene expression

The standardized transcriptome data were used to cluster *C. humilis* leaves under different alkali treatment times. Heat maps were drawn using TBtools.

### 2.9 Protein interaction prediction

The GATA protein interaction network was constructed by STRING 11.0 (https://string-db.org/cgi/input.pl). Using *Arabidopsis thaliana* as a template, the network edge was set to confidence, the parameter was set to a medium confidence parameter (0.400), and no more than 10 interaction factors were displayed.

### 2.10 Transcriptional activation assay of *ChGATA14* by *ChWRKY29*

*MdWRKY115* is an important regulator of osmotic stress and drought stress tolerance in apples. *MdWRKY115* can bind to the W-box elements of the promoters of downstream target genes to regulate their expression and participate in response to osmotic stress and drought stress. In this study, ChWRKY and MdWRKY115 were used to construct a phylogenetic tree, and *ChWRKY29* was screened out. The *ChGATA14* gene, which was significantly up-regulated under alkaline stress, was screened by transcriptome data for gene function verification.

The full-length coding sequence (CDS) of *ChWRKY29* was inserted into the pGreenII 62-SK vector as an effector, while the w-box promoter fragment of *ChGATA14* was cloned into the pGreenII0800-LUC vector to generate a reporter gene. Aspergillus tumefaciens strain EHA105 was used to transfer all construct vectors. The effector and reporter were transiently co-infiltrated into 4-week-old tobacco leaves. A. tumevalis cells were cultured in suspension buffer (10 mM MgCl_2_, 10 mM MES, 150 mM acetosyringone, pH 5.6) until OD 600 was 0.6. After 2–3 d of injection at 23°C, tobacco leaves were sprayed with water as a control. Fluorescence in tobacco leaves was observed and photographed using an automatic chemiluminescence imager (Tanon 5200, Shanghai, China).

## 3 Results

### 3.1 Identification of the *ChGATAs*

Using the *Arabidopsis thaliana* protein sequence as a reference, we screened *ChGATA* using the BLASTP comparison method. Following HMMER, CD-Search, and SMART analysis, a total of 20 *ChGATA* genes were pinpointed and designated as *ChGATA1-ChGATA20*, reflecting the chromosomal arrangement of *ChGATA*. The complete coding sequence (CDSs) of the *ChGATA* gene spans 403–1657 base pairs. The physicochemical characteristics of 20 ChGATA proteins were then examined in more detail.

The lengths of sequences range from 131 (ChGATA19) to 542 (ChGATA20) amino acids, and their molecular weights extend from 14.286 (ChGATA19) to 60.238 (ChGATA20) kDa, indicating considerable structural differences, potentially associated with genome size and species evolution. The isoelectric point falls within the range of 4.83 (ChGATA1) to 9.69 (ChGATA5). Among these, 12 (60%) ChGATAs exhibited a pI below 7, indicating a notable prevalence of acidic amino acids in most ChGATAs. **Table 1** encapsulates the essential elements of the ChGATA family. As expected, a majority of ChGATA proteins (18 out of 20) are located in the nucleus, suggesting a major function of ChGATA in regulating nuclear transcription. Two ChGATA proteins are located in chloroplasts. Considering that their functions may be involved in the physiological and biochemical regulation of chlorophyll synthesis.

### 3.2 Chromosomal localization of genes and tertiary structure analysis of GATA family gene proteins in *C. humilis*

**Figure 1:**
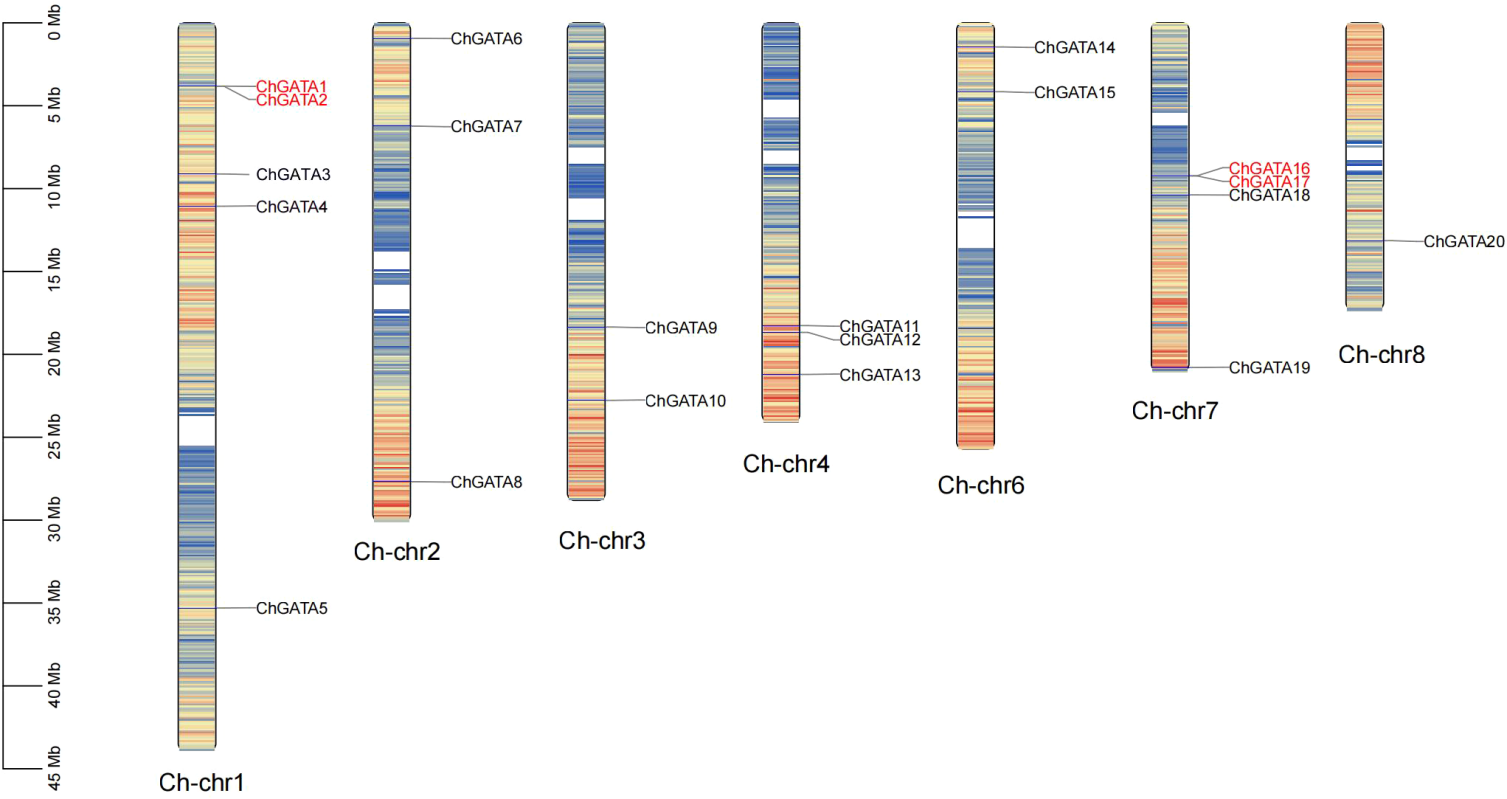
Chromosomal positioning of *ChGATAs*. Each of the 20 *ChGATAs* is displayed on the chromosomes, denoted by their names. The left scale illustrates the C. humilis chromosomes’ length. Numbers of chromosomes are shown at each bar’s base. The red lines on the chromosome indicate a dense distribution of genes. The genes marked in red font are tandemly repeated genes.

**Figure 2:**
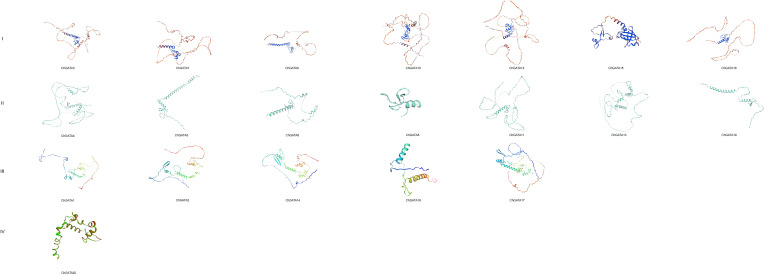
Predicted tertiary structure of ChGATA family member proteins.

To delve deeper into the chromosomal distribution of the *ChGATA* gene, We mapped the locations of each member of the ChGATA gene family in the *C. humilis* genome, discovering 20 *ChGATAs* situated on the respective Chr1-Chr8 chromosomes (**Figure 1**). Except for Ch-chr5, all other chromosomes contain *ChGATA*. Ch-chr8 contains only one gene (*ChGATA20*), and Ch-chr3 and Ch-chr6 both contain two genes (*ChGATA9–10* and *ChGATA14-15*). Ch-chr1 contains the most *ChGATA*s, up to five (*ChGATA1-5*). The distribution of genes on *C. humilis* chromosomes varies, possibly indicating disparities in their size and structure. The ChGATA gene family consists of two tandem repeat genes (*ChGATA1* and *ChGATA2*, *ChGATA16* and *ChGATA17*), which are located on Ch-chr1 and Ch-chr7 respectively (**Figure 1**), both from subfamily III. Fragment replication may be the main driving force for the expansion of members of subfamily III. The three-dimensional structure analysis of GATA protein in *C. humilis* showed that there is a significantly larger protein structure among different members (**Figure 2**).

### 3.3 Evolutionary study of the ChGATA

**Figure 3:**
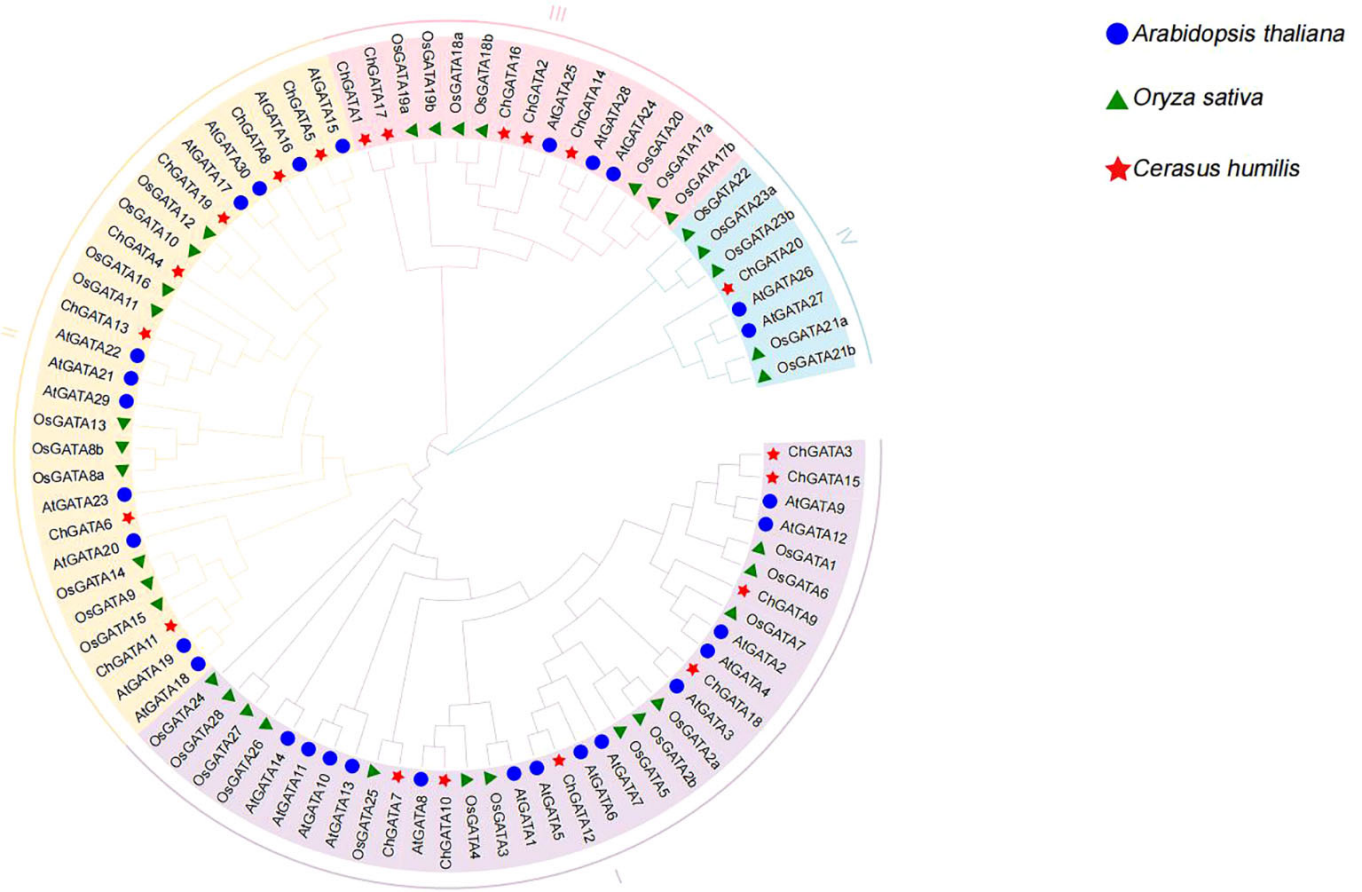
The phylogenetic analysis of GATA in *Arabidopsis thaliana*, *Oryza sativa*, and *C. humilis* by the NJ tree includes 30 GATAs from *Arabidopsis thaliana*, 28 from *Oryza sativa*, 20 from *C. humilis*, constructed using entire protein sequences.

To construct the phylogenetic tree, the amino acid sequences of 20 identified ChGATA proteins, 30 AtGATA proteins, and 28 OsGATA proteins were utilized. (**Figure 3**). Based on AtGATAs’ categorization, there were 20 ChGATA proteins segmented into four distinct subfamilies (I, II, III, and IV). Among them, the most ChGATA members are Subgroup I and Subgroup II, both containing 7 members, followed by Subgroup III, containing 5 members, and finally Subgroup IV. Evolutionary analysis revealed that several ChGATA proteins were closely grouped with AtGATA, indicating that their homology was close and their gene functions might be similar.

### 3.4 Collinearity and evolutionary analysis of genes

**Figure 4:**
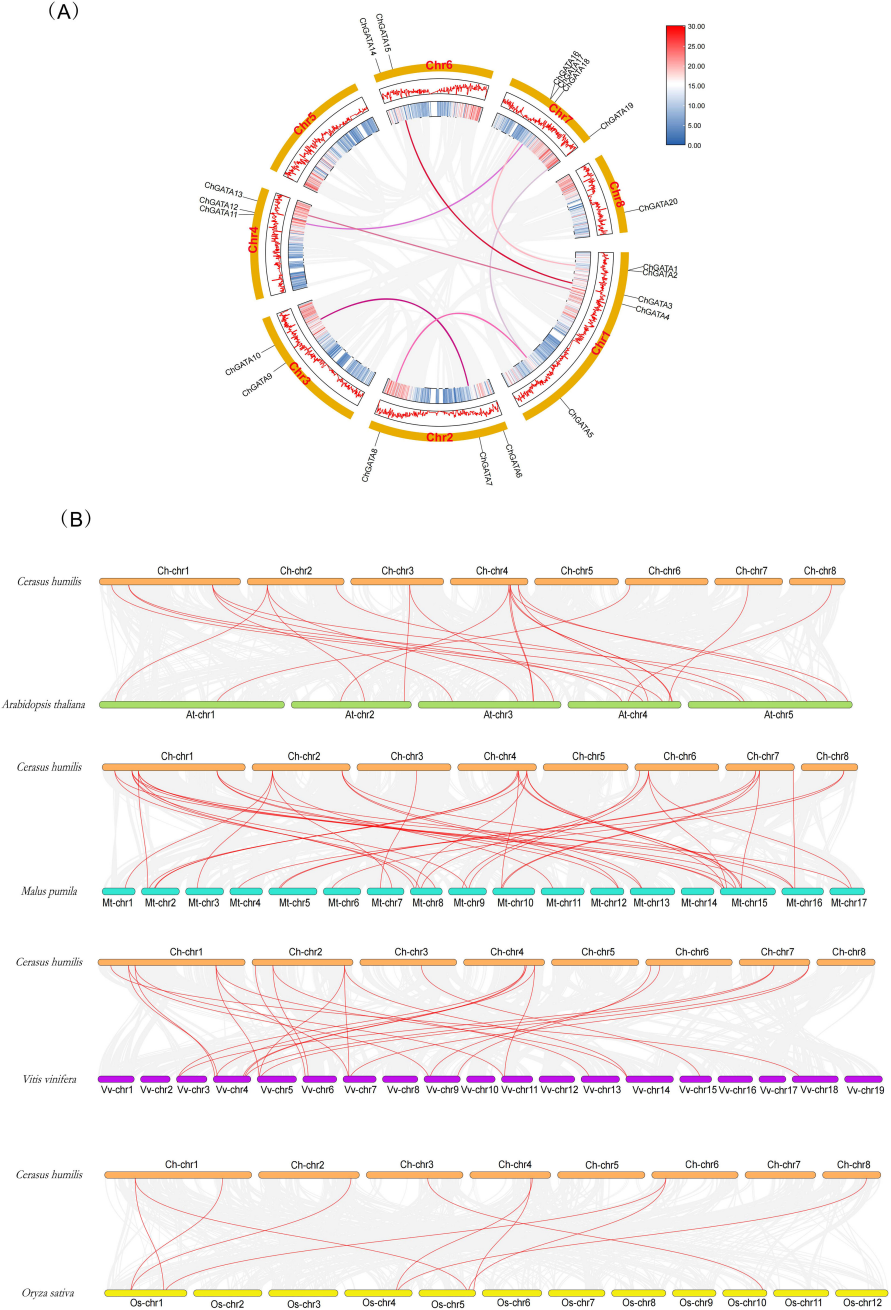
Locations of chromosomes and their Synthetic examination. **(A)** Locations of chromosomes and the segmental duplication occurrences in 20 *ChGATA* genes. These segmental duplications are marked by red lines. **(B)** Examination of *GATA* gene synthesis in the genomes of *C*. *humilis*, *Arabidopsis thaliana*, *Malus pumila*, *Vitis vinifera*, and *Oryza sativa*. The red lines indicate gene pairs that are homologous between two neighboring species.

Tandem and segmental duplication events can expand the number of gene family members, which is an important driving force for gene family amplification and the main driving force for species evolution (Altschul et al., 1997) (**Figure 4**). The MCScanX method was used in this study. It was found that there were 7 fragment repeat gene pairs on the repeats of *C. humilis* genome (*ChGATA1* and *ChGATA16*, *ChGATA3* and *ChGATA15*, *ChGATA4* and *ChGATA13*, *ChGATA5* and *ChGATA19*, *ChGATA5* and *ChGATA8*, *ChGATA7* and *ChGATA10*, *ChGATA12* and *ChGATA18*). Among the seven gene pairs exhibiting fragment repetition, three *ChGATA* gene pairs are part of subfamily I, another three from subfamily II, and one from subfamily III. Subfamily I and subfamily II may have mainly expanded through fragment replication during the evolutionary process.

To gain a deeper insight into the genesis and development of GATA family genes, we conducted a collinear examination of various species, encompassing monocotyledonous species such as *Oryza sativa* and dicotyledonous ones like *Arabidopsis thaliana*, *Malus pumila*, and *Vitis vinifera*. A total of 23,43,26 and 10 collinear *GATA*s were identified. There was a higher count of collinear genes in dicotyledonous plants compared to monocotyledonous plants, with *Malus pumila* (rose family) having the most collinear gene pairs, perhaps it is because both of them originated from the Rosaceae family. These results may provide valuable reference for gene function in Rose-family cash crops.

### 3.5 Analysis of gene structure and protein motif

**Figure 5:**
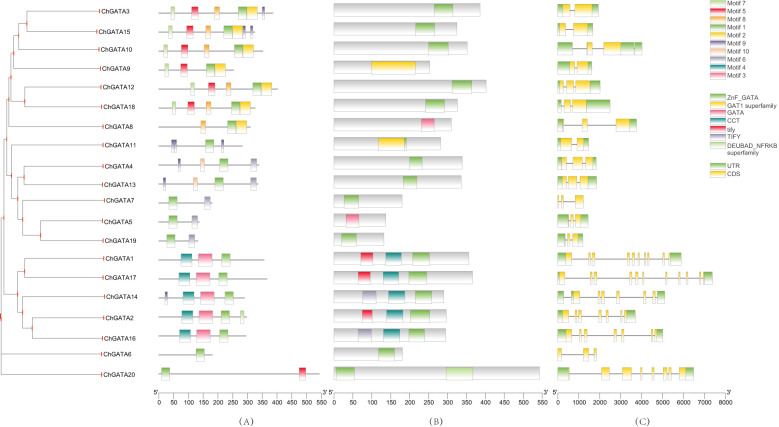
Analysis of *ChGATA*s’ phylogenetic makeup, gene architecture, and preserved motifs. **(A)** Conserved motifs of ChGATAs, with black lines denoting the length of the protein sequences. **(B)** Conserved domains of ChGATA. Up/down stream sequences are denoted by black rectangle **(C)** The genetic structure of ChGATA. The UTR (untranslated region), CDS (coding sequence or exons), and introns are symbolized by green rectangles, yellow rectangles, and black lines, respectively.

For the purpose of examining *C. humilis* GATA’s evolutionary diversity, the preserved patterns of 20 ChGATA proteins were scrutinized via the MEME website, and a composite diagram of phylogenetic trees, motif patterns and gene structures of 20 kinds of ChGATAs was constructed using TBtools (**Figure 5**). The *ChGATA* has between one and ten introns; the majority of genes have two introns, but the *ChGATA1* and *ChGATA17* genes have several, suggesting that the *ChGATA*’s introns may have been lost during evolution (**Figure 5**). Furthermore, genes grouped into subclass may display distinct structural characteristics. For instance, *ChGATA3* in subfamily I has two CDS and two UTR fragments, while *ChGATA6* and *ChGATA8* in subfamily II do not have UTR regions.

In the study, a total of 10 conserved motifs were identified, together referred to as Motif1-10. Most members clustered in the same subclade have similar motif structures, suggesting that members in the same subclade may have similar functions. A basic portion of the GATA domain known as motif 1 is present in every member of the ChGATA gene family. Members of Class II lack Motif 2, in contrast to Class I members who possess Motif 1, Motif 2, and Motif 8. The variance in the quantity and nature of preserved motifs in *C. humilis* GATA proteins mirrors their structural variety and suggests distinct biological roles.

### 3.6 Analysis of cis-acting components

**Figure 6:**
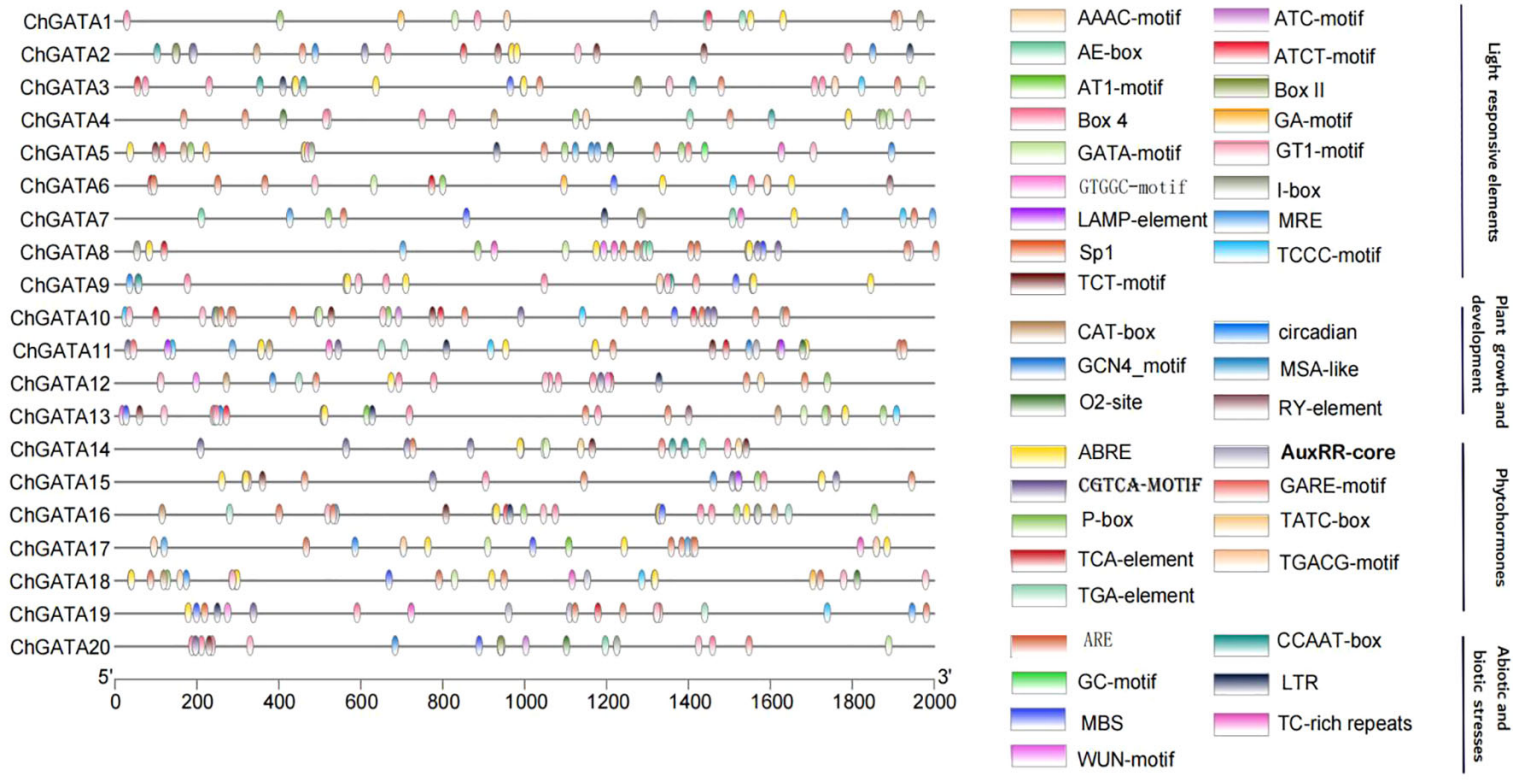
Forecasting a cis-acting component in the 2000 base pair promoter located before the 25 *ChGATA* genes.

Non-coding DNA segments known as cis-acting elements, located in gene promoter areas, play a crucial role in controlling the transcription of related genes. In this instance, a specific area situated 2000 base pairs before the *ChGATA* gene’s transcription initiation point was chosen as the hypothetical promoter region (Yao et al., 2022). Subsequently, the *ChGATA*’s promoter sequence was isolated and entered into the PlantCARE database to identify cis-acting elements. Findings indicated that out of the 20 *ChGATAs* promoter sequences, the highest count of photoresponsive elements was 263, the most prevalent among all promoter sequences. This was succeeded by 75 abscisic acid response elements, prevalent in most promoter sequences (**Figure 6**). Within the domain of elements linked to environmental stress, the primary components of *ChGATAs* are the hypoxia-inducible element (GC-motif), low-temperature response element (LTR), and drought–inducible element (MBS). The majority of *ChGATAs* contain two hormone-related elements: ABRE, TGACG-motif, and CGTCA-motif. The majority of these genes include photoreaction-related Box 4 (ATT AAT) and Sp1 (GGG CGG), which are crucial for regulating photoresponse.

### 3.7 Analysis of *ChGATA* gene expression under alkali stress

**Figure 7:**
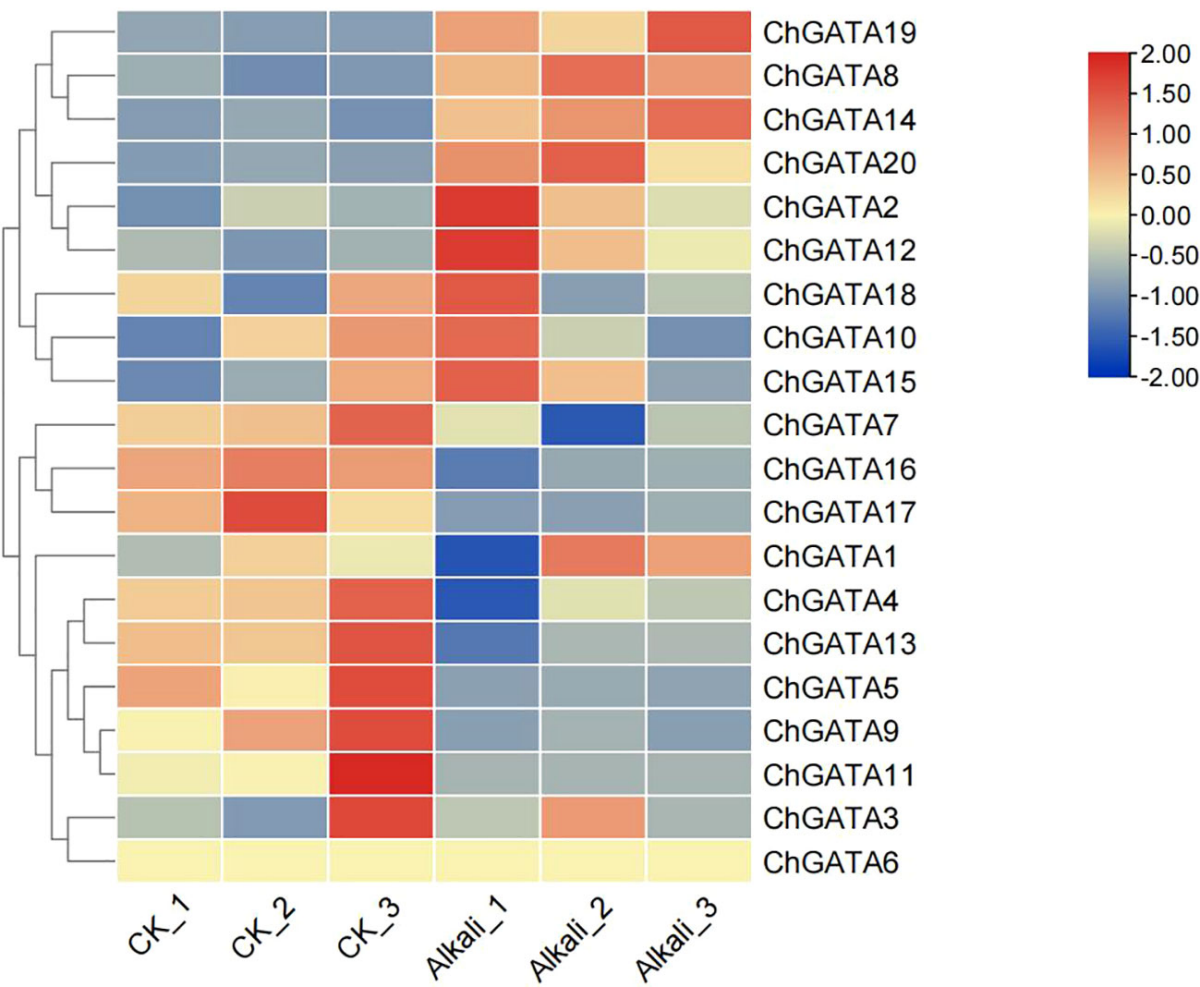
Pattern of *ChGATA*s expression under *C. humilis’* alkaline stress. This pattern, derived from FPKM plus 1 post log2 transformation, was examined using heatmap hierarchical clustering.

An investigation into the gene expression in *C. humilis* leaves was conducted to understand the *ChGATA*s’ function under alkali stress (**Figure 7**). Findings indicated alterations in *GATA* gene family members’ expression in *C. humilis* leaves post-alkali treatment versus the control group, with distinct gene response processes. For instance, genes like *ChGATA2* and *ChGATA12* were elevated due to alkali stress, and their levels rose under alkali stress, hinting at their potential role in resistance to alkali stress. In contrast, alkali stress led to a reduced expression of *ChGATA1*, *ChGATA4*, *ChGATA7*, and *ChGATA16*.

### 3.8 RNA extraction and quantitative RT-PCR analysis

**Figure 8:**
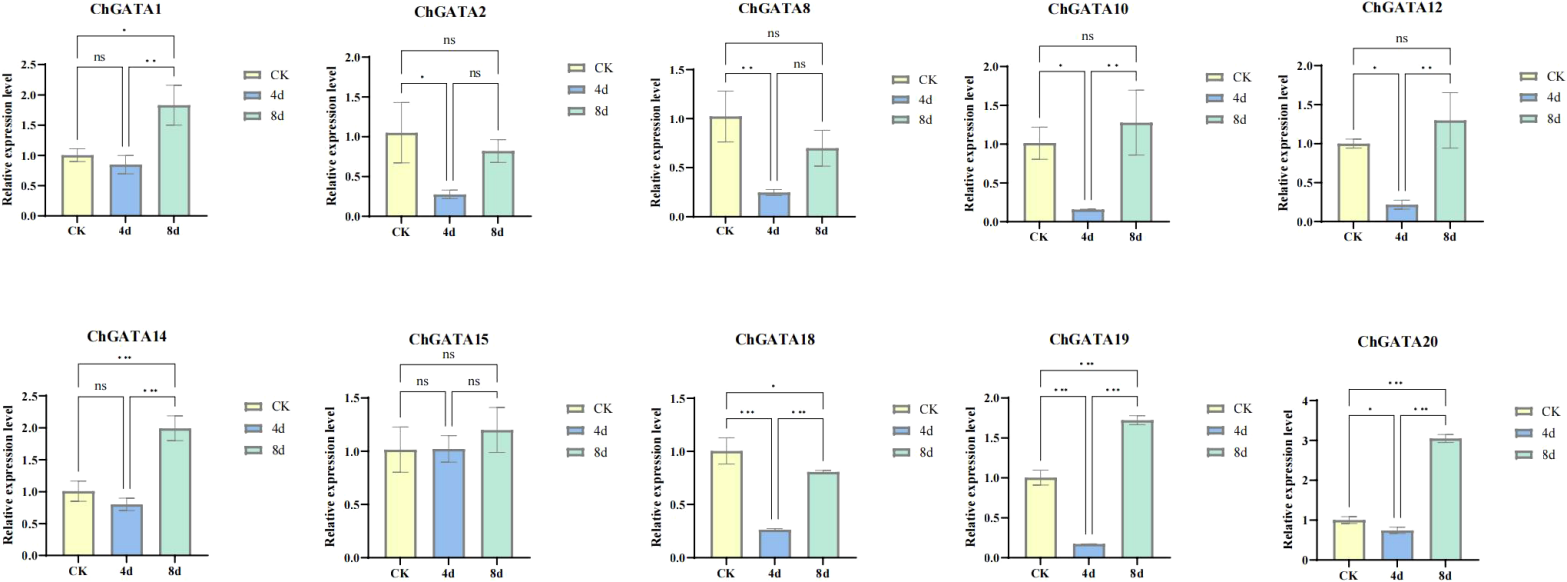
The qRT-PCR examination of *ChGATA*s under alkali stress reveals a statistically notable variance from the control group (p* < 0.05; p** < 0.01; p*** < 0.001). ns indicates no significance.

**Figure 9:**
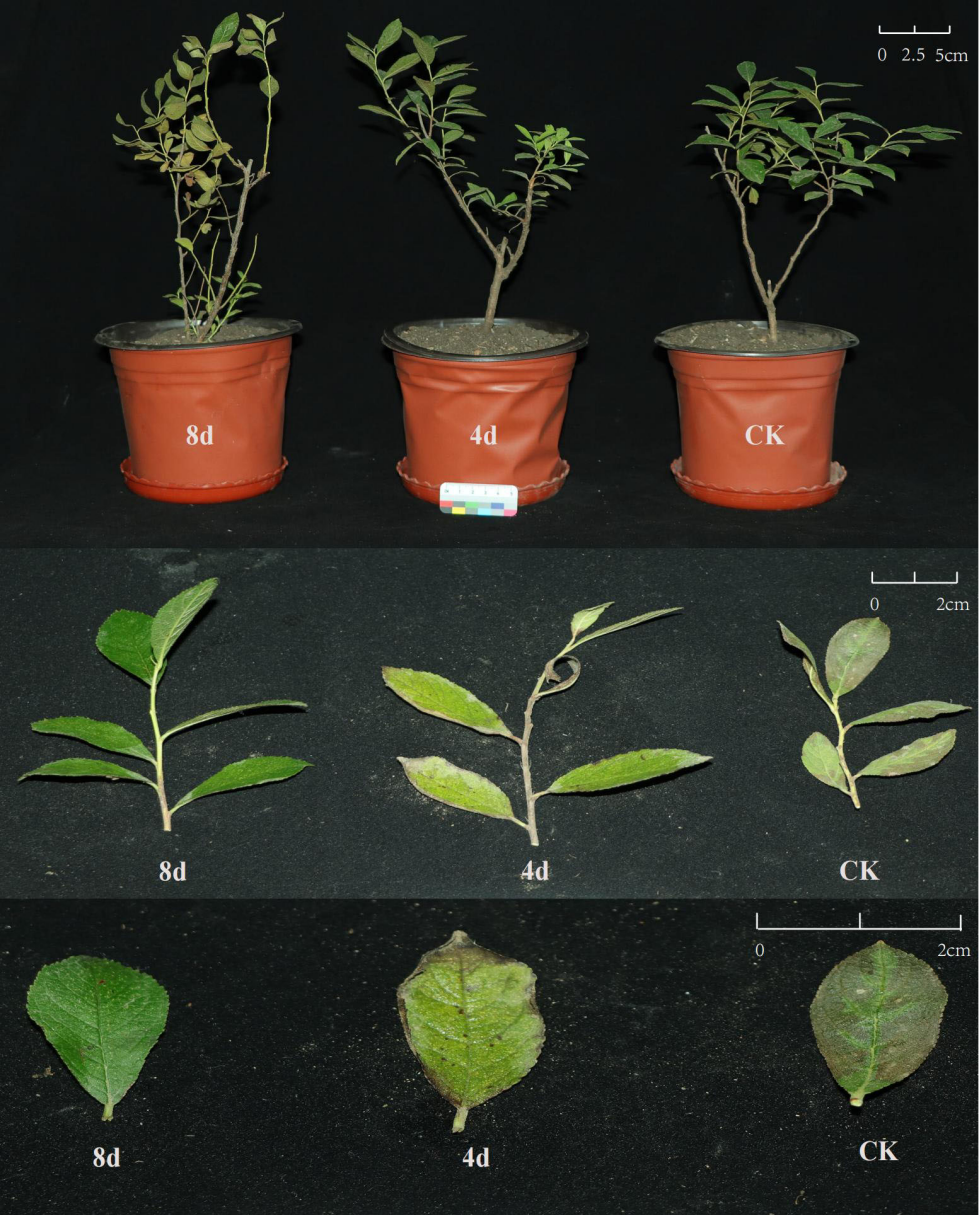
Plant diagram of *C. humilis* under alkali treatment.

To further understand the functions of *ChGATAs*, we selected 10 representative *ChGATAs* based on the heat map. The analysis was conducted using qRT-PCR technology, and the results are shown in the **Figures 8**, **9**. Compared with the blank control group, the expression levels of *ChGATA2*, *ChGATA8*, *ChGATA10*, *ChGATA12*, *ChGATA18*, *ChGATA19*, and *ChGATA20* were significantly decreased at 4 days of alkali treatment. The expression levels of *ChGATA1*, *CHGATA14*, *CHGATA19* and *ChGATA20* significantly increased after 8 days of alkali treatment. Most *ChGATAs* show a trend of first decreasing and then increasing.

### 3.9 Protein interaction prediction analysis

**Figure 10:**
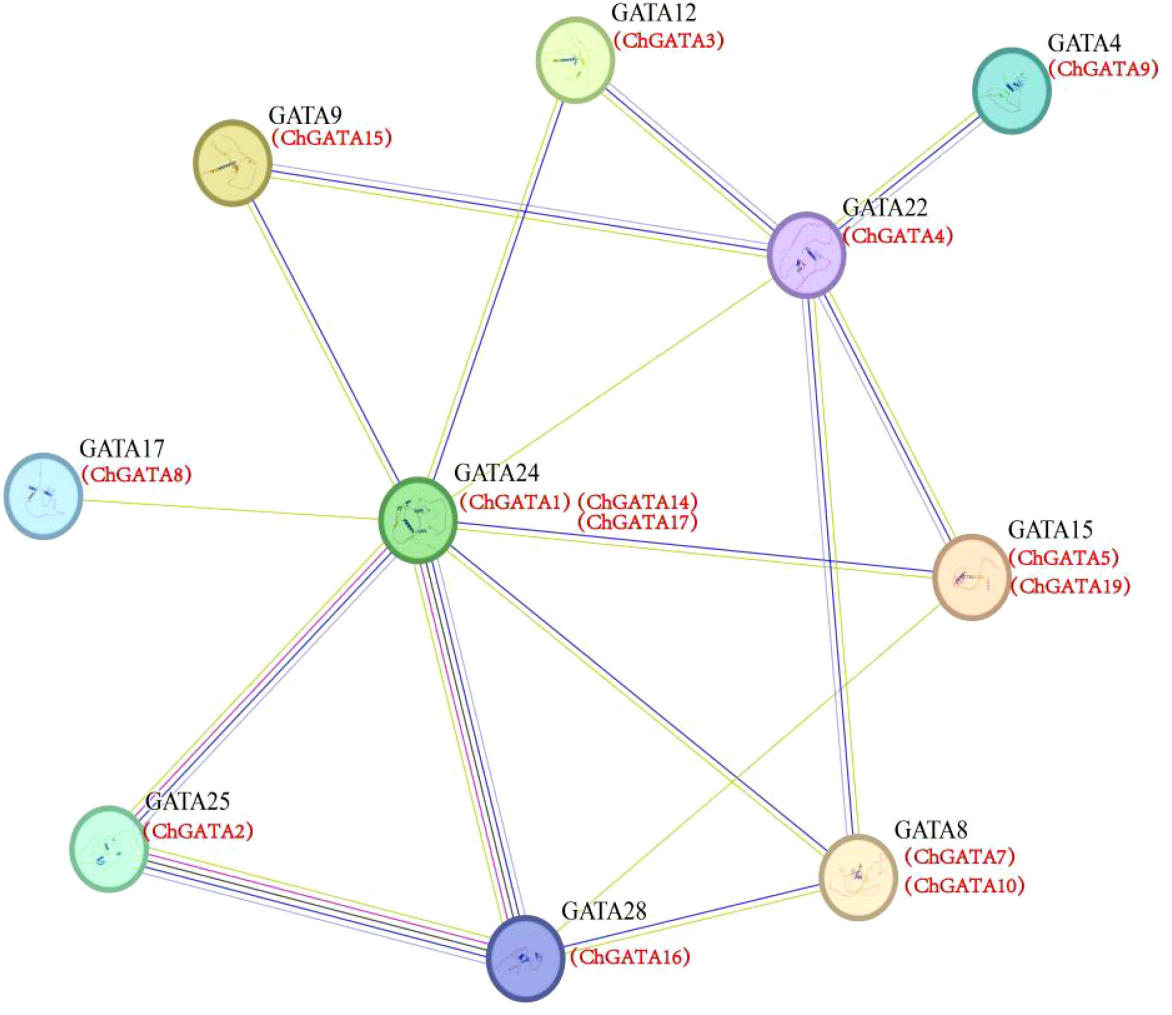
Protein interaction prediction analysis.

To explore potential regulatory interactions between ChGATA proteins, a protein interaction network was constructed using the STRING database (**Figure 10**). The results showed that the 14 ChGATA protein interaction, among them, the ChGATA1, ChGATA14, ChGATA17 has more protein interaction, and 10 other protein interactions. In contrast, ChGATA8 and ChGATA9 each have only one interacting protein. It is worth noting that ChGATA1 ChGATA14, ChGATA17, ChGATA2, ChGATA16 interaction effect is stronger, they all come from the family III, prediction of ChGATA protein interaction networks could help to understand the function of the connection between these proteins.

### 3.10 Transcriptional activation assay of *ChGATA14* by *ChWRKY29*

**Figure 11:**
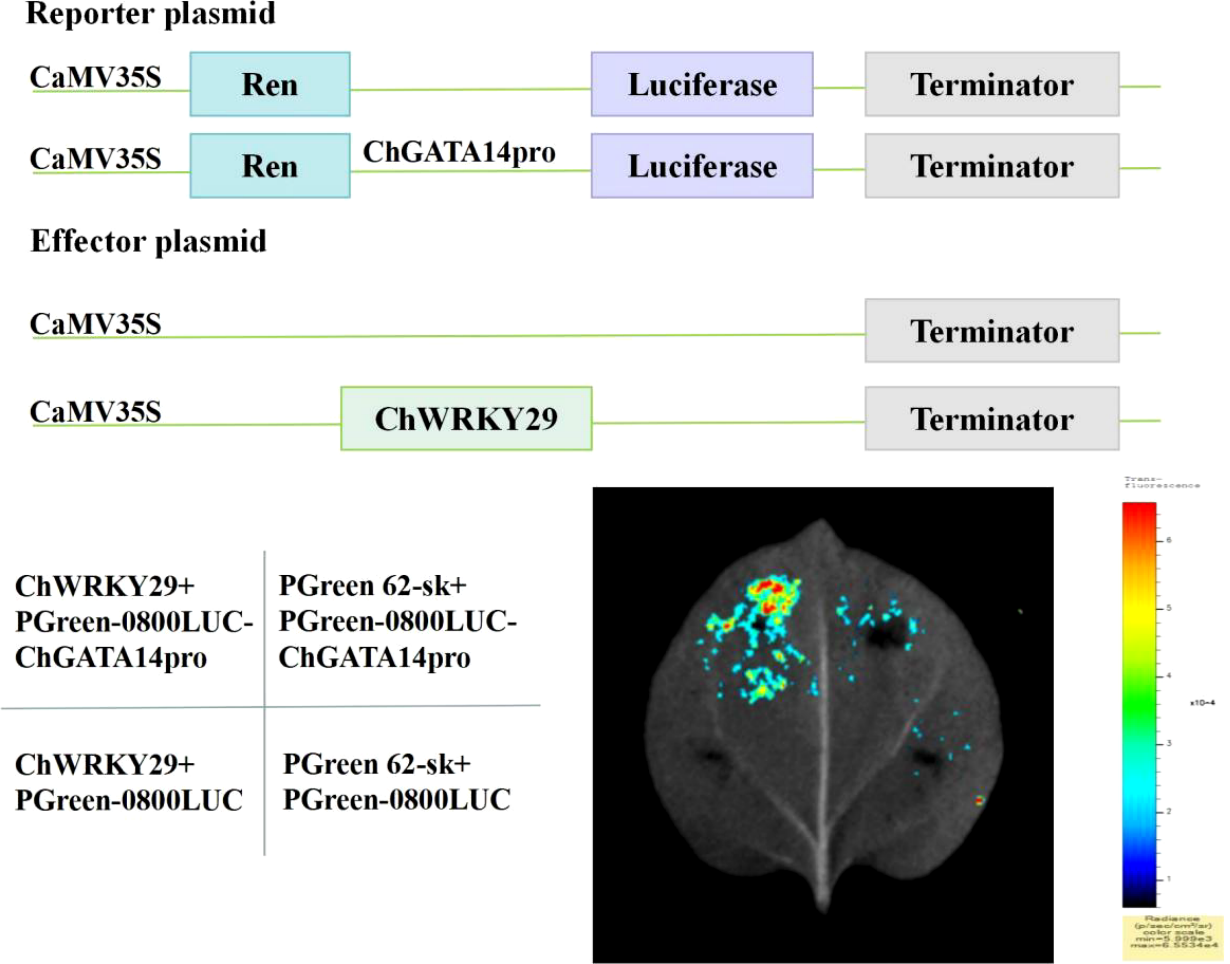
Dual luciferase imaging experiments, including plasmid construction, injection site, and imaging results.

In the present study, we performed a dual luciferase reporter (DLR) assay in tobacco leaves (Nicotiana benthamiana) (**Figure 11**). The relative intensity of the LUC signal was significantly increased after cotransformation of *ChWRKY29* with pGreenII0800-LUC-ChGATA14-w-box compared to the vector control, indicating that *ChWRKY29* activated *ChGATA14* expression. Thus, DLR analysis confirmed that ChGATA14 may be transcriptively activated by ChWRKY29 to resist alkali stress.

## 4 Discussion

*C. humilis* is nutrient-rich and is known as the third generation fruit with high nutrition and high health care value together with sea buckthorn and blueberry, which has attracted more and more attention from researchers. Transcription factors in plants, including *WRKY* (Liu et al., 2019b), *MYB* (Li et al., 2019), *bHLH* (Geng and Liu, 2018), and *Bzip* (Zhang et al., 2016), are crucial in controlling genes involved in diverse biological functions related to plant growth, stress reactions, and hormone communication routes (Franco-Zorrilla et al., 2014). However, in recent years, various abiotic stresses have affected the growth and development of *C. humilis*. These environmental challenges not only hinder the physiological processes necessary for the survival of *C. humilis*, but also affect the economic vitality and conservation value of *C. humilis*. The GATA gene family plays an important role in improving plant stress tolerance and breeding, but to date, no study has been conducted on the GATA genes in *C. humilis* (Reyes et al., 2004). In this study, a total of 20 ChGATA family members were identified in the *C. humilis* genome, a number close to that found in four other Rosaceae species, including peach, indicating that *GATA* genes are relatively similar in number in the same genus (Manzoor et al., 2021). The striking differences in protein length, structure, molecular weight, and theoretical isoelectric points among ChGATA members suggest that GATA would generate functional divergence to participate in different physiological processes during evolution. For example, *AtGATAs* with consensus sequences of different lengths separately regulate various physiological responses such as hypocotyl growth, root development, flowering, lateral root formation, root branching, and cell differentiation. Subcellular localization predictions suggest that *ChGATAs* function primarily in the nucleus, consistent with findings in *Cucumis melo L*, *Glycine max*, *Cucumis sativus*, and *Solanum tuberosum L* (Zheng et al., 2024).

Based on the GATA family grouping in the model plant Arabidopsis thaliana, the 20 *ChGATAs* were grouped into four subfamilies: subfamilies I (7), II (7), III (5), and IV (1). This is consistent with most GATA family studies, suggesting that the GATA family is relatively stable during evolution (Liu et al., 2020; Zhang et al., 2023). Analysis of the conserved motifs of the 20 ChGATA proteins identified motif 1 in all 20 ChGATA proteins, suggesting that this conserved motif is required for ChGATA protein function and is the zn-finger GATA motif of the family. Motif 3 corresponds to the CCT motif, and motif 7 to the TIFY domain. CCT and TIFY domains play important roles in flowering and root development in Arabidopsis thaliana, suggesting that members of groups I and III may have similar regulatory functions in *C. humilis* (Zheng et al., 2024). Gene structural information provides clues to the evolution of gene family members. Consistent with most plant GATA family members, groups I and II have 2–3 exons, whereas groups III and IV have 7–11 exons. This further indicates the relative stability of GATA family members in plants. Conserved motifs/gene structures are distributed similarly across subfamily members, yet significant variances among subfamilies suggest precise subfamily classification, aligning with prior study findings (Manfield et al., 2007; Yu et al., 2019). The majority of GATA TFS proteins in plants, like those in *Arabidopsis thaliana*, *Oryza sativa*, *Triticum aestivum*, and *Vitis vinifera*, possess a solitary zinc finger domain (Reyes et al., 2004; Zhang et al., 2018; Feng et al., 2022). Nonetheless, subfamily III members include extra domains like CCT and TIFY. Earlier research has noted this and demonstrated its crucial function in various biological processes, such as regulating embryonic and flower growth, stress resistance, and diverse plant hormone signaling pathways (Richter et al., 2013b; Behringer and Schwechheimer, 2015; Gupta et al., 2017).

Gene replication fosters the creation of novel genes and their roles, significantly influencing evolutionary processes. It manifests in three primary forms: fragment replication, tandem replication, and translocation events, with segment replication and tandem replication being prevalent in the expansion of plant gene families (Zhu et al., 2014; Wang et al., 2019). Two tandem repeat gene pairs (*ChGATA1*, *ChGATA2*, *ChGATA16* and *ChGATA17*) were identified among the 20 *ChGATAs*. Tandem repeat genes may replicate during evolutionary divergence in plants and maintain their copy number at a high frequency. Analysis of the fragment repeatability of the 20 *ChGATAs* on the chromosome identified seven pairs of duplication events, mostly belonging to subfamilies I and II. This indicates that these genes are linked in their respective subfamilies and that most of the gene structural and functional information is retained after the evolution of whole genome duplication. These results indicate that genes in different subfamilies differ in structure, function, environmental adaptation, mutation, and recombination. In addition, 20 *ChGATAs* were used to analyze interspecific collinearity with monocot and dicot plants. The results showed significant collinearity family members between calciocarps and dicots between GATA family members. Only a few GATA members are collinear with monocots. Due to chromosomal localization, fusion, and selective gene loss, specific *GATA* genes may fail to localize to any synteny region, making chromosomal synteny difficult to identify (Yang et al., 2008).

Acting elements within the promoter region are involved in regulating gene expression by binding to trans-acting factors to regulate the activity of target genes (Liu et al., 2019a). The presence of these elements suggests that ChGATA family members are involved in various functions such as growth and development, stress response, and hormonal regulation. The W-box element is a DNA cis-regulatory element that is specifically recognized and bound by the WRKY family of transcription factors. In terms of abiotic stresses, such as low temperature, drought, and salinity, WRKY transcription factors also bind to W-box elements to regulate the expression of downstream stress resistance genes and help plants resist stress. For example, the *ShWRKY33* promoter in wild hairy tomato contains W-box elements, which can induce downstream gene expression and improve the low temperature resistance of tomato under low temperature stress (Guo et al., 2022). In this study, based on MdWRKY115, which has been reported to play an important regulatory role in stress in apple, and combined with the transcriptome differentially expressed genes, *ChWRKY29* and *ChGATA14* were selected to respond to alkali stress in *C. humilis*. *ChWRKY29* can activate *ChGATA14* expression to resist alkali stress. In addition, transcription factors usually regulate various reaction processes through protein-protein interactions. This study predicted potential interactions among 20 ChGATA members. The results showed that ChGATA14 had strong interactions with ChGATA2 and ChGATA16, and ChGATA14 was a differentially expressed gene that was significantly up-regulated under alkali stress. It may play a regulatory role by forming protein complexes.

Previous studies have shown that the *GATA* gene has a distinct function in plant development. Generally speaking, gene expression patterns can offer crucial hints for predicting gene function (Zhang et al., 2019). In this study, the expression of 10 *ChGATAs* was analyzed based on RT-qPCR under alkali stress. The results showed that the expression profiles of most *ChGATAs* were first decreased and then increased with the treatment time, indicating that lye could affect the expression of *ChGATA* gene, and some upstream regulatory genes might be activated at the early stage, which inhibited the expression of *GATA* gene. As the stress continues, the expression of these upstream genes may change, or some other compensatory mechanism may be activated, which releases the inhibition of the *GATA* gene and activates the regulatory factors related to *GATA* gene expression so that *GATA* gene expression is increased to participate in the subsequent stress response process and regulate the expression of downstream genes related to stress resistance (Gupta et al., 2017). These results can only be used as a reference, and further studies are needed to understand the specific functions of these genes. In conclusion, this study provides a new idea for the study of stress resistance of *C. humilis* and an important theoretical basis for the selection and improvement of calcaneus fruit in the future.

## 5 Conclusion

This research methodically pinpointed and examined the GATA TFs gene family in *C. humilis*, classifying twenty *ChGATAs* into four distinct categories across seven chromosomes. While class I members had Motifs 1 and 2, class II members lacked Motif 2. Fragment duplication was the main driver of the *GATA* gene family’s proliferation in *C. humilis*. Transcriptome heat maps show that, the *ChGATA*’s expression is distinct under alkali stress, with the same *ChGATA* gene subgroup exhibiting varied patterns. qPCR analysis showed that most of the screened *ChGATAs* were first decreased and then increased with time, suggesting that these genes were initially repressed and subsequently turned on a compensatory mechanism. In addition, the dual luciferase assay and the predicted protein interaction structure showed that *ChWRKY29* could activate the expression of the differentially expressed gene *ChGATA14* in response to alkali stress, and ChGATA14 was at the core of the protein interaction network and had a strong interaction with ChGATA2 and ChGATA16. These results suggest that *ChGATA14* may play a role by forming protein complexes. However, this gap in the validation of *ChGATA* function needs to be filled in future studies using knockout/overexpression experiments to confirm the role of key *ChGATAs* in alkaline stress response.

## Data Availability

The original contributions presented in the study are included in the article/**Supplementary Material**. Further inquiries can be directed to the corresponding author/s.
