# Supplementary material for: Genome-wide identification, phylogenetic and expression pattern analysis of GATA gene family in Cerasus humilis
Source: Front Plant Sci. 2025 Jun 5;16:1596930. doi: 10.3389/fpls.2025.1596930 (PMC12176744; doi:10.3389/fpls.2025.1596930)
Supplement: Supplementary file 2 [file Table2.doc]

>Chumilis18605.1 + Up_Stream_Len 2000

gtccaagtagatgaagggccaaatagtaaaatacactagtcctcgaaatttaatgtcagg

ttttgtatgaggaaactatgcggctttacaaatttcacctgctggtggaagttgtattct

atggtgagacatgtgtgcccttctttcacagtgatggatttgtaacataggatatacaga

ttatttgtcagtcgtcacctctctctcataggaaaatgaatatttcctcttagccatttg

ggaatggatgaacctttgtaatttcatggaggaatgttttgttttttcttttcccccctc

tatttgacatgtcaatgacactcatcatttgtaagcccaattgaataaatgacttgatgg

gcagaaattacttattgaatatttccagaccataagtgctctctctctctctctctctct

ctctctctctctctctctctctctctctctgcatgcgttctcttgctgctgaagttgcaa

gtttttttaaagggagttgttattgacagttaaataaagtcatctagtactcagaatgca

atttctccgtcatatttttggaatgctaagaacaacttcccctttttgtagttaacaggt

ggatcgtgtgatggtcatatagttcttaggatcatatatccaccaaatgggttttgcttc

ggttgttcttttgagattttggttgacataggtgaggtgacgatagaacttggttttaaa

actccctacatgcacggctctagaagttgaaccgatcacagtgttattaagacgagtgaa

ggtatttgacacatttgctaacacataatttcatgaacatatgtcaataacattctacag

tgtttttatgttgacgattgattatatatttctaatcttgactaatttaccaacacatct

aatgaatgcgatccaatgactgagattagaaatatgtaaccattcttaatgcccatttga

cacattagctaacacgtcatttaatgaacatattagttatccgagggtcgaagacatttt

acaattttattaagataagaataaggtatttgacacattaactaacacattatttggtaa

acaaatttggttatccaaagtctggtattcgaccttgccatatcccaaaataggtcagtt

tattaaagtcttaccttcatgaaaaaagtcacaagtcagtttattattattattattatt

attattttataaaagagaagccagttttctaatgctatataaacatgttttgttatttct

ttaagaaaccctaaatctaatggattgaggacaaatcttcaattctcgaaatacctgaca

acagaaggcctccgcacgatcccaccgttgatcataaaaggcgctcacttcaatccaacg

gccacacatcttcgttcttcatctctacccgaattttcaagtttctcgtgttctgttttt

tctgctgtacatcgtttggaatcacaaaccggaatcggaattaattattccattctcgga

taccgctatcccagttctgaattatcttaccctcgaacggtaattccgatgatgtaaaat

ttccaatttttttttctccatttcgtattgaaatttctgtttgcttgcccagaaacgtaa

ggaaaattatggaaatgttcaattactaatttcaactaagcaaatcactctcacttatac

atgcaactccatttcgtacaattctgtcatgatttcttattattttttcttaattttcct

gcattttctaggcaatcaaacacagtaatactaccttgaactaaatacatcatttgcgtg

tattagcacagttctctgcaatttttatcataattttgtttccctgcattttcctggcaa

ccaaacagagcactgagccatttgatctttgaattgatagctttagcattgacatgaagg

tgaaaagcagtaaatttacacaagtttgttcaaaggagtagtgatcttggaagagtgtga

tacaaattgagtagtgcgca
