# Supplementary material for: Genome-wide identification, phylogenetic and expression pattern analysis of GATA gene family in Cerasus humilis
Source: Front Plant Sci. 2025 Jun 5;16:1596930. doi: 10.3389/fpls.2025.1596930 (PMC12176744; doi:10.3389/fpls.2025.1596930)
Supplement: Supplementary file 4 [file Table4.docx]

cds

>Chumilis00510.1 -

ATGGCGGCCGTGAATCCGCAGCCACTGCAAGCGCGACCATTCGAGGAGCACGGGAGAGGT

CCGATACCGATCGAAGACGATGAGGCTGAGTACGAAGATGGTGGTGATGATGGTATGGAG

GACACGGAAGAGGTTCATGTGAATTCGGTCAGCGTTGCGGAGCGTGGAGGAGGAGGAGGA

GGAGGAGGAGGAGGAGGTGTGGTTATGGCGTCCAGGACTAGTGAGCTCACTCTGTCTTTT

GAAGGCGAGGTCTATGTCTTCCCTGCTGTTACACCTGAGAAGGTGCAAGCAGTACTATTG

TTATTGGGAGGACGTGATGTGCCAACTGGTGTGCCTACAGTTGAAGTGTCTTACGATCAG

AATACACGGGGTGTGGCCGACACCCCAAAACGTTCAAATCTTTCACGAAGAATAGCTTCG

CTGGTTCGGTTTCGTGAAAAACGGAAGGAGAGATGCTTTGACAAGAAAATTAGGTACACA

GTCCGTAAAGAGGTTGCACAGAGGATGCTTCGTAAAAATGGACAGTTTGCATCGTTAAAA

CAAAATTCAGGTGATTCTGGTTGGGATTCGGCACAAAGTGGCCTTCAAGATGGCACTTCT

CGACCAGAAACTGTCTTACGGCGATGTCAACATTGTGGAGTTAGTGAAAATAATACTCCT

GCAATGCGCCGTGGACCTGCTGGCCCAAGAACCTTATGTAATGCATGTGGTCTTATGTGG

GCGAATAAGGGAACACTGAGAGATCTCAGCAAGGGAGGAAGGAACCTTACCATGGACCAT

ATAGAACCTGGAACACCAATCGAAGTGAAGCCTTTACTTGTTGAAGGAGAATTTTCTGGA

AACCAGGATGAGCATGGAACACTTGAAGGTTCTTCTAAAACAGTTATTGAAAGATCCAAT

GATGCTTCTGTCAACCTGGATGAACAAGATTTGCATGAAACTGCTGAAGATCTTACAAAC

AGTTTGCCGATGGGGATTGTTTCCTCAGCCAATGATGAGCAGGAACCTCTGGTTGAGCTC

ACTAATCCTTCGGATACAGATTTAGACATCCCTGCTAATTTTGATTAG

>Chumilis00511.1 -

ATGTACGGACACTCCGAGCCCATGACCATACCCAACCCGATCCCCGCCGGTGGCGACGAC

GATGCCGCCGGCCCCGGTGTCGACTCCATTGACAACGCCCACATTCACTACGAACCCCAT

ACGCTCGAGGACGGCGGCGGCGTCGTCGCTGTCGTCGAGGATGTAAGCTCCGACCCCGTC

TACGACGTCGGTAGCTCCGAAATGCGTGCTCAGCCTTACGACGGCTCCAGTCAGCTCACG

CTCTCGTTTCGTGGCCAGGTCTTTGTTTTTGACGCTGTTACGCCTGAGAAGGTTCAAGCA

GTGCTATTACTGTTGGGGGGTAGCGAATTATCTTCCGGCCCTCAAGGTGCGGAGTTGGCA

TCTCAGAATCAGAGGGGTACAGAAGATTTCCCCATACGGTGTAGTCAGCCTCACAGAGCA

GCGTCATTAAGTAGGTTTCGTCAAAAGAGAAAAGAGCGATGCTTTGATAAAAAAGTCAGA

TATAGCGTCCGCCAAGAAGTTGCACTCAGGATGCAGCGTAACAAGGGGCAATTTTCTTCT

TCGAAAAAGTCAGATGGAGATTATAGCTGGGGTAATGGCCAGGAGTCAGGGCAGGATGAT

AGCCATGCAGAAACCTCCTGCAAGCATTGTGGAATAAGCTCAAAGTCCACACCAATGATG

CGGCGTGGGCCATCTGGTCCAAGGTCTCTTTGCAATGCTTGTGGGCTTTTTTGGGCAAAC

AGGGGGACTTTGAGGGAGCTTTCCAAGAGAACCCAGGATCATTCTGTGACTCCAGCTGAG

CAGGGTGAAGCTGATACTAAAGATTTGAACAGCGTAACTGCCATCGATGCACACAACAGT

CTTGTTCCCTTCTCTAATGGTGATAGCTCAGCCTTAGTTGCTGAACAGTAG

>Chumilis01338.1 -

ATGGAAGCTCCCGAGTATTTTCAGAACAGTTTCTGCCCACAATTCCCACCGGAAAAGCGC

CACTCTTTTGACAATAATAACAATAAGGCCACCAATGGCGGTGGCTGTGGCGGGGACCAT

TTCATGGTGGAGGACCTTCTCGACTTTTCCAATGACGACGCGGTGATAACCGACGGTGGT

ACTACTTTTGATAACGTCACCGGAAACTCCACTGACTCTTCCACCCTCACCGTCGTCGAC

AGCTGCAATTCTTCTTCCTTATCGGGCTCCGAACCCAATGTCATCCCCGATATCGGGTCC

AGAAATATCGCCGAAGGCCCATTCTCCAGTGACCTCTGCGTCCCGTACGACGATTTAGCT

GAGCTCGAATGGCTTTCGAATTTCGTGGAGGAGTCGTTTTCCAGCGAGGACCTGCAGAAG

CTGCAGCTGATATCAGGAATGAAAGCCCGACCCGACGAGGCAGCTTCCGAGACCCGACAA

TTCCAACCCGAACCCAACCGAAACGACAACGCTCACAACACCACCACCACCAACAACAAC

CCGATATTCAACCCGGACGTTTCGGTACCCGCCAAGGCCAGAAGCAAACGGTCCCGGGCC

GCCCCATGCAACTGGACCTCCCGCCTCCTCCTCCTCTCCCAGCCGACGTCGTCCTCGGAC

CAATCCGACGTCGTTTCAAGTGGGCCGGCGTCTCCGTTACAGCCGCCTTCAACCACCGGG

AAGAAAACGGTGAAGTCGGCGCCGAAGAAGAAGGAGAGCCCTGAGGGTCCGGGAGGAGGG

CCGGGGGACGGCCGGAAGTGCCTGCATTGTGCGACGGACAAGACGCCGCAGTGGCGGACG

GGGCCCATGGGCCCGAAGACGCTGTGCAACGCGTGTGGGGTCCGGTACAAGTCAGGTCGG

CTCGTACCCGAGTACCGACCCGCCTCGAGCCCGACGTTCGTGCTGACGAAGCACTCCAAC

TCGCACCGCAAGGTGCTGGAGCTTCGGCGCCAGAAGGAGATGGTGAGGGCGCAGCAGCAG

TTTATTCACCAAGTACCGCCGCAGCAGCATCACCACCACCACCATCATCACCATCAGAAC

ATGGTTTTCGATGTATCCAATGGTGGAGATTACTTGATTCACCAACACGTGGGGCCCGAC

TTCCGGCAGCTGATCTAG

>Chumilis01670.1 -

ATGATGACACCAGTGTATCTGAACCCAGCATCCTCTCCTTTCTCCATGGTGGAGCAAGCA

GAAGATCAACGCCTAAAGCTCTTTATATCACCACCTTATGATGAAGCTGCTTCATGTACT

TCTTTACCATTTCCTACTTTCTTCGACTCCTTGCAAGATCAAACACCTGGGACTACTTTT

ACTTCTCTTGCTCATCATCAATCACAACTATATCATCACAAGGATAAGAACATTTGGGAT

TGCGGAACAAGTTACGATCAAGCATCCTCATCATCTTCTTTAGTTCAAGCTCATGTGGTG

GACGCTATAAGCAACAAGGACCGTAGATTGTCCAGGTGTGGTGATCATGAAAGAGAGACC

AATATTGGAGGAGAAGAGGAAGGTAAAAGTAGTAATTATATCACCAGGCCAAGGTCAGTG

AAGTGGATGTCCTCCAAGATGAGGTTGATGCAAAAGATGACCAGCAACAACCCTGATCTC

CCACCTGGTACCACTGATCATATACCAGCTGAGATTTCTGAGCATAAATTCCAAATTCAT

GCTCAGCCTCGAGAAATTAGCGAAACCAGCTTTTCCAGTAATAGCAACAACACTGCGGCT

GTTAGGGTTTGTTCTGATTGTCACACCAACTCAACCCCACTTTGGAGGAGCGGCCCTCTC

GGTCCCAAGTCACTGTGCAATGCATGTGGCATTCGGCAGAGGAAAGCGAGACGAGCCATG

GCAGAGGCTGCGGCTGCGGCTGCAAATGGGTTTGCTGTTGGCAGCGCCGACACTTCATCT

CCAAGGGGTAAGGTGGCCAAGGAGAAGAAATCGCGGGGCAGCCATAAGAACAAGATCAGC

AAGCTCATCATCACCGACAATGCATCAATATCTCACAATAACAAAAAGAATAATAATAAC

AAGAAGAAGATTTGTTTCAAGGCTTTGGATTTTCAACGAGTGTTTCCTCAGGATGTTGCT

GAAGCTGCAATGCTCCTAATGGAACTCTCTTGTGGCCTAATCAATAATCACTCTTGA

>Chumilis04435.1 -

ATGTTGGATCCAAGCGATAAAGGATCAGAGTCCGAGGAGATGAATATCAAAACTCCAGAT

GTGGGTTCATCCGAAGAAGGCCATAAGAAGACCTGCGCCGACTGCGGAACCTCCAAAACT

CCTTTATGGAGGGGAGGCCCAGCAGGCCCTAAGTCGCTATGCAATGCGTGCGGGATCAGA

AGCAGGAAGAAGAGAAGGGCCATTCTGGGCTTGAACAAAGAAAACCCCAATGATAAAAAG

GGCAAGAGGAACAAGCAGCTAGGCGATGGTTTGAAGCAGAGGCTATTGGCTCTGGGAAGA

GAGGTTTTGATGCAGAGATCGACGGTGGAGAGGCAGAGGAGGAAGCTAGGGGAAGAAGAA

CAAGCGGCTGTGCTACTGATGGCTCTTTCATATGGCTCTGTCTATGCTTAG

>Chumilis05700.1 +

ATGGATCCCAAAGGTGTTCAAAATGGTTTCGAAATGACCAACTTTGATCAACATGAAGCC

AATATAGGAGGTACGGATTGTTTGGTGGACCTCACCCTGAGGCTAGGAATACCATCTTCC

GACAAAAACAATGATCAACAATCCCATACTGCAAATGGTTCTTCTACCTCTCAGGCCGTC

GATAGGTCTGGCCTCAACAATCTCAACGTTAATGGATACAGGCAATATGAATTTCCAGCT

CCACCGGAGTTGAAGAATTACTGCATAATAAACATATCCAACAGAAGAGGAAAAACTGGT

GGCTCGAGAAAGAGGAAGACTACTGGACGCCGTCCAGCTAAAGTTGGTGATATCGACAGA

ACCTGCACCAACTATAACTGTCGTGTTACCGAATCTCCCATGTGGCGTACTGGTCCCCTT

GGCCCCAAGAGTCTTTGCAATCGGTGTGGGATCAGGTTCCGAAAGATAAAGCAAAAAGAA

GAAACAGAGCAGCAGGCTGCGGTAGCAGCTTTCAAACAGCTTCATGCGGTCTCCAAGCTT

TAG

>Chumilis06509.1 -

ATGACGGCGGTCTCTGCTGACGTCACCAAGCTAACGAAGCGTGAGAATATTAGTGCCATG

GCTAGTTCTATGCCCACTGATGATAATTTCAATATCGAGAGCTTCCTCCCTGACGAACTC

AAGGCTCTTGATTTCCCCATGGAAGACATTGAAGAGTGTGTTGAAGACGAAGATTGGTAT

GCGCAATTCCAAAACCTCGAACCGCCTTCCTTTGAAGATTTGTTTTGTCCAAGAGTTTTG

GATGGTAATGACGACATGAAACCTAAGAACCTCTCCACTTCGTATGGACAAACTTCTCAG

CGAAATCAATTACCAAGGACTGCTGCTAAAACTACCCGAGGCATAAGCAGTATTCGCAGC

GAGGACCTTGAAAGTATTGGCTCTTGTGCTGCTCAATTTGAGCCCAAGTTCGAAAAGCGT

ACCCGAAGCAAGCTTTCACGAAGCAAGCGTTCAAGCCCTGCTATTTTCAATACTCAGTTC

TTCCCCAGCACCTCATCCAACTCCTCCGCCTCTGAAAATTTATATAATTGGGATGCTTCT

GAATCAGACTTAGAAGGCTCTCTCACTGAGGAGATGTCGAACGCTGCCAAAAGAAAACAA

AAGAAGAAAAGGAATCTATCGCAGCTCTCAAGTGCAGAGATGAGAAAGCTCTCCACGGAG

GAACCAGGTGAGAGTCGGGAGACTAAAAGATGCATGCACTGTGCAGTGACGAAGACCCCT

CAGTGGAGGGAAGGGCCACTAGGACCAAAGACCCTCTGCAATGCGTGCGGGGTTCGTTAT

AGGTCTGGCCGTCTCTTTCCGGAGTACCGCCCTGCTGCAAGTCCTACATTCGTTGCGTCG

GTCCATTCCAACTCCCACAAGAAGGTTATAGAGTTGAGAAACAAGGGTTGCCAGGGGGCT

ACTATGGGAATTTTATCCTCGGCCCAGTAG

>Chumilis08509.1 +

ATGATGAATTGGGGGAGAAGATATGTGGCTTTAGATAGTGTTTTGGGGAGAAAGGAACCA

TTTCCTCAAGCTATGAATGGGAACAAGAAGCGCTGCTCTGACTGCATGACCACTGAGACA

CCCTTGTGGAGAGGTGGCCCAGCCGGGCCCAAGTCACTGTGCAATGCATGTGGGATCAGA

CACAGGAAGAGAGGAATTCCTACTGTGAGCTTGATGAGCAAAGCGCCAAAGAGGAGGAGA

GAGAAAACATGTGGCGGAAGTAGCAGTACTATCACAACCACATATAATATTGGTGCTTCT

GCCACACACAATAATGCTTCTGCCACAACAGCCAAAAGCACTTTTGGTAGTGGTGGTGGT

GGCATAAATTTGAATGAGCCTCCTAAGGTGAGGTTTGTGGGTTATGGTGAGAAGGTGTTT

TTGCAAGACTCGCAGGCAGAGGGGGAGGGGGAGAAACAGAGCCAGTGGAGGGAGTGGGGA

GAGGTGGAACAAGCAGCTGTGTGTTTGCTGGCTATGTCATGTGACTCTGTTTTTGCATGA

>Chumilis10293.1 +

ATGGAGGACGTGTACGGAGGAGGAGCATCTGCTTCCTCAGCCCAACAACAGCAAGACTAC

TTTCCCATTGACGACCTTCTCGACCTGTCAAACGACGACCTTTTCTCTTCCTCCACTTCC

TCCACCGACTCCATTGACCTCCACCCTCCGCCGCCACCTCCGCATCTCCACGTCAGTTCC

ACCGTCTTCAACCCCACCGCCGCCACCGACTTCACCAACGACCTCTGTGTACCCAGTGAT

GACGTGGCGGAATTGGAGTGGTTGTCGCGCTTCGTGGACGACTCCTTCACCGACTTCCCC

ACCACCAACGTCTTCGGCTCCGCGTCCTTCCCAAACGACACGTCGTCCTTGTTTCCGAGC

CGGGTTCGCACCTATCGCTCAAAATGGGGCGGCCCACCCGAGCCCAGCGACTCACGGGCG

AAGCCCAAGAGAGAGCCATCGGAAGCTTCTCCGTCGCCGTCCAAGCCGCGCCGGTGCGCG

CACTGCGCCTCGGAGAAGACGCCGCAGTGGCGGGCGGGTCCAATGGGCCCCAAGACGCTG

TGCAACGCGTGTGGGGTCCGATTCAAGTCGGGTCGGCTCGTGCCCGAGTACCGACCTGCG

GCAAGCCCAACGTTCGTGCTGACTCAGCACTCCAACTCGCACCGGAAGGTTCTGGAGCTC

CGTCGCCAGAAAGAGGCTTCTCAGCAGCAGCAACCCGAAGAGCAACACCAGCAGAAGCAG

CAATTCTATCTTCACCGGGATGAGTATCAAGTGTGCTGA

>Chumilis11028.1 +

ATGATCGGACCGAACTTCATCGACGAGATAGACTGCGGGAGCTTCTTTGACACCATCGAC

GACCTCCTCGACTTCCCAAACGACGACGTCGAGTCCGGACTGGGCCCCGCCCCTGACTGC

AACGCTGCGTTTAACAACTCCCTCTGGCCCAACCAGTCCGGCTCGCTCCCGGCTCCCAAC

GACGCTGTTTTCTCAGCCGGCAACTCCGCCTCCGACCTCTCCGCCGAGCTCTCCGTTCCG

ATTGAAGACATAGTTCAATTGGAATGGCTGTCGAACTTTGTTGAGGATTCCTTCTCTGGT

GGAAGCCTCACCATCAACAAACCAGACTCCTTCATCAACAAGGACACATCCCACCACCAG

TTCCGGACCTCCAGCCCAATTTCTGTGCTTGATAGCAGTAGCTCTTGCTCAGGTGACAAG

AATGTGCCGCAAAGTCCTGGACCGGTCGCTGCTCCCGGTAAGCGTGGACGTGCTCGCAGC

AAGCGTCCACGCCCGGCCACCTTCAACCCTCGCTCCGCAATTCAACTCATTTCCCCTGCT

TCCTCTGTTACTGAGGCAGAGGGGCCCCAGGCTCAGCCATTCCTTGCCCCAAAGGCCCCT

TCTGATTCTGAGAATTTTGCAGAGTCTCGTCCTGTGATCAAGATACCAAAACAAGCTTCT

GGGGAGCAGAAGAAGAAGAAGAAGCTCAAGGTGTCGCTTCCCCTGGCTCCTTTGGACGGG

AATCAAAATTCCGGTCCTACACAGGCAGCAGTGAGAAAATGCTTGCATTGTGAGATTACT

AAGACACCCCAGTGGAGGGCAGGCCCAATGGGACCGAAAACCCTATGCAATGCCTGTGGC

GTCCGCTACAAGTCAGGCCGGCTCTTTCCTGAGTACCGGCCTGCTGCCAGTCCTACTTTT

GTTCCATCCTTGCACTCCAATTCCCATAAGAAGGTTCTTGAGATGAGAACCAAGGGTGGC

GAGTTGGTTGTTTTTGGAGAGACTGCAACAGCTATGAACGAAACTCCCGAACTCATTCCA

AATACTAACAGCAGCATCTCCATGGATTACATGTGA

>Chumilis14463.1 +

ATGCAGAGGTGTAGTAGCAGCTCACATGGGAACATGACCGGTCCATGTACATGCGGTGTC

CTCCACCACACCCAATCCAATTCCTCCTTCTCCATGCTCTTCTCCATGCCCAACCATCAC

AAGCCGTACGATCATCATCATCATCACCATCACGAAACGCAGCATGATCATTATAATCAC

ATGTACCCGTTCGCATCCTCTTCCTCCTCTGTCGACTGTACTCTCTCGCTAGGCACTCCC

TCCACCCGCCTCACCGAAAACGACGTCATCCTCGACGACAAGCGCACCCGCAACGAGCGC

CGCAGCGTTTCCAACTTCTGCTGGGACTTGTTGCAGCCCAAGCACCACGCCACGTCAGCC

ACGTCTTCCCACCATCACAAGAACGGCAGCCACCGCAGCGGCGGCAATAGTAACGGCGTA

TCTAACGCCGTCCACTCTAATAACGACCCCCTCCTAGCCCGCCGCTGCGCCAACTGCGAC

ACCACCTCCACCCCTCTCTGGAGAAACGGTCCAAGAGGCCCCAAGTCACTGTGCAATGCC

TGTGGAATTCGGTTCAAAAAAGAAGAGAGGCGTGCCACGGCGGCTGCAGCAAACGGGGCC

AGTTCGAGTGTCGTAGGAATGGAGCACAACAGCCACATGTTGAGCCAGCACCACCACAAC

AATTCATGGATGCCGCATTCACAAACCCAGAAAATGCCATGCTTCTCTCCTGCCATGAGC

AACGAGTTTCGGTTCATGGAGGACGACACTGCGCACCACGAAAACGACGCCACCGGCATT

CCCTTCCTCTCTTGGCGTCTCAACGTCACAGACAGACCAAGCCTCGTCCATGACTTCACA

AGATGA

>Chumilis14552.1 +

ATGCTTTACCAAACTCACCACTCCTTTTTATTCCAATTTCATCCCTTTACTTGTTCAATC

CCTTCTTCTTCTTCAACCACCTCAACCTCAACCTCAACCTCTCCCTCTCCCCTCTCCTCT

CTCTCTCCTCATCCTCCTCTGCTCTCAACCCGTCCTCCACCTCTACCTCCACAACAGGTT

GGAACTGAAATGGAATGCGTGGAAGCGGCCCTGAAGACCAGTATTAGGAAGGAGATGGCT

GTCAAGGCGAGCCCACAAGCGGTTTTTGATGACTTGTTGTGGGGCGGCGTCAATGGGCAA

AACGGTGTCGCTTGTGACGATTTCTCAGTCGATGACCTCCTTGACTTCTCGAACGAAGAT

GGGTTTGTGGAAACAGAGGCTGAGGAAGACGATAAAGATAAAGTTAAGGGCTTTGCCTCT

GTTTCGCCTCAGAAACAACCCCAGGACCCTGAGAACTCGGATTTGTCTGAGAAAAACGAA

CTTGGGCCCGAACCCACCAGCGAACTCAGCGTTCCGGCGGACGACTTGGAAAACCTCGAA

TGGTTGTCTCATTTTGTTGAAGATTCTTTCACGGAATTCACTACGTCTCTGCCGGCAGGG

TTCATCCCCGAGAAGCCCAAGACCGAGAAACGTCCCGACCCGGCAACGCCTTTACCCGAA

AAACCCTGTTTCAAGACTCCGGTTCCGGCCAAGGCCAGAAGCAAGCGAACCCGAACTGGC

GGCCGGGTCTGGTCACTGGGGTCACCTTCGTTGACGGAGACATCCTCGAGTTCTAGTTCC

TCGTCTTCTTCTTCGTCTCCGTCGAGCCCCTGGCTCATTTACGCCACCACCCAAAACCGG

GAACCGGCCGAAGCGGGCGGCGAACCGGTCGGGTCAGTGGAGAAGCCTCCGAAGAAGCCC

AAGAGAAGACTGGTGGATGGAAGCTCGAGCCAGCCGCCTCGTCGGTGCAGCCATTGTGGG

GTGCAGAAGACCCCGCAGTGGAGAACCGGGCCCAATGGAGCCAAGACCCTCTGTAACGCC

TGTGGGGTCCGTTACAAGTCGGGTCGGCTCTTACCGGAATATCGACCCGCATGCAGCCCG

ACTTTCTCGAGCGAGTTGCACTCCAACCACCACCGTAAAGTTCTAGAGATGCGGAAGAAG

AAGGACGTCACCGGCGTGCCCGAACCCGGTTTGACCAGACCTCCGGTGGTGCCCAGTTTT

GGTTAG

>Chumilis15041.1 +

ATGGCTCCACCTTCCCCTTTTCTTGAGCTCAGTGGAGATGGTGGAGATCATGATCATCAA

TACCACCATCTCTTCAATCTAGAACCTCAAACTTCTTTCTCTTCTTCCTCTCTTTCCAGC

CCCCTATTCTTAACTCCATCTCAAGCCCAAGCACCATCCGATCACTATAGAGAACCACAA

AACTTTCAATTCCAACTCCTAGAGGCTGATCATCATAACATTGTCTCGTATGGCGGATCA

TGTGATCACGACCCTCAAACCCTCGAAAACGAAAGCGGCAGTGGTACTATTCTGAAATTA

AGCATTTCCAAGAATGGAGCTGGCAGAAATGGAAATCCACGTACTGATAAGTGGATGTCT

TCAAAGATGAGGATGATGAAGAAGATGACAAACCCGGATAAAACTTCGAGCAGCTGTACA

CCCAGCGATGATAAACCAGTTGCAATGAAGTTATCAATATCACACAAGTCTGAAGAGCAG

AAGCCGCAGCATCCTGACATGATCAGCTGCAGCAACAATTCATCCAACAACATGAACAAC

AACGTCCCAATTATTAGGGTTTGCTCTGATTGCAACACTACTAAGACACCTCTATGGAGA

AGCGGACCGAGAGGCCCCAAGTCACTTTGCAACGCCTGTGGAATTCGGCAAAGGAAGGCA

AGGCGGGCCATGGCAGCTGCTGCTGCGGCAGCAAGCGGCACAACCCTGGCTGCCGCGCCA

TCTATGAAGAGTACTAGTAAGGTGCAACACAAAGACAATAAACCAAGAGTTGCTTCTACT

GTCCCGTTCAAGAAAAGGCCTTACAATAAACTCAGCTCAACCCCACCATCAAAAGGCAGA

CCACCAAAGAAGCTTTGTTTTGAGGATTTCGCAATAAGCATGAACAATAACCATTCATCA

TCTGCCACTACCACAACCACTACTAGCCTTCAACGAGTTTTCCCACAAGACGAGAAGGAA

GCTGCGATCCTGCTCATGGCTTTATCTTGTGGACTTGTTCATGGTTGA

>Chumilis18605.1 +

ATGGATGACATCTGTGGCAGCGATGAACCCATGCATATGAATGATAATCTGCATTTGCAA

TACATGCAAGAGCATGAGCATCATCACGGGTTGGACAATATAAGCAATGTAAATGGGGTG

GCTGATGATCATGATAATGGAAGTGGTGGAGCTGAACTTGTCCAAGCCGATGTCCCATCT

GACCCCATGAATCTTTCTGATACGCATGATGGGATGATGGACCATGGCCCCGAAAATGGG

GACCAGCTCACATTGTCGTTTCAGGGTCAGGTTTTCGTGTTCGACTGTGTGTCACCTGAG

AAGGTTCAAGCTGTGTTGTTACTATTGGGGGGCCGTGAAGTACCTCCAAGCATGCCTGCT

GTCCCAGTAACCACGCAGCATGCTAATCGGGGCCTTACTAGTACTCCACAACGGTTGAAT

GTCCCTCAAAGACTGGCTTCACTAATTAGATTCCGTGAAAAACGGAAAGAACGAAATTTT

GAGAAGAAGATTCGTTATACTGTTCGAAAAGAGGTAGCACTTAGGATGCAACGAAAGAAA

GGTCAATTCACATCTTCTAAACCCAATAATGATGATTCTGCATCAGCTGTCACAAGTTCG

GGCTCGAACGAGAGCTGGAGTCAGGATGGTAATGGATCCCAACATCAAGAGGCTGTCTGT

CGACACTGTGGCATCAATGAGAAGTGTACACCAATGATGCGACGTGGACCTGATGGACCA

AGAACCCTTTGCAATGCCTGTGGACTTATGTGGGCAAACAAGGGTACTCTGAGGGACATC

TCCAAGGCAGCAGCAGCAGCAGCAGCAGCACCCCAAGCAGGACAAAACCCTTCCATAAGC

AAGAACGAAGATATGAAACCTGATTTATAA

>Chumilis19001.1 +

ATGGAAATGCCGGAATTCTACCTCGGCGGCTACTTCGACGCCGGAGCCGCCGAATTCCTG

CCGGAAAAGAAACCCGCCGCGGCTGCCGGCGATCAATTCACCGTCGACGACCTCCTCGAC

TTCTCCAACGAAGACGCTCTAGTCGCCGACGGCGGGTTCTTGGACGCCGCTGCCGATTCC

TCCGCTGTCACCGCCGTAGACAGCTGCAATTCCTCGGTTTCCGGGGGAGAGCCTCAGTTC

TCCGGCAACCGGAGCTTCGGCGACTCTCAGTTCTCCGGCGACCTTTGCGTTCCGCACGAC

GACTTGGCGGAGCTAGAATGGCTATCGAACTTTGTGGAAGACTCCTTCTCCGCAGAGAAG

GACTTGCAAGCTCTCCAATTCCTGTCCATGACCACAACCAAACCTCAAACCCCCGAAACG

TCCTCGTCCTCTGAAACGAATCAAAACGCACCGCTTTTCCACCACGAAACGCCTCTCCCG

GGAAAAGCCCGAAGCAAGCGCTCACGCGCTGCCCCCGGAGACTGGTCCACGCGCCTCCTC

CACCTCATCACTCCCAATGACACCATCAAGCCTCCCAAAACGACAACGTGCAAGAAGAAA

GATGGTATTGGTGGCAATTCGAACTCGGGTTCCGACTCCTCGGGCCGGAAATGCCTCCAT

TGCGCGGCAGAAAAGACCCCGCAATGGCGGACTGGCCCCATGGGCCCAAAAACACTTTGT

AACGCGTGTGGAGTTCGCTATAAGTCCGGCCGGCTTGTGCCCGAGTACCGGCCCGCCGCG

AGCCCCACTTTTGTGTCGGCTAGGCATTCCAATTCGCATAGGAAGGTTTTGGAGCTCCGG

AGACAAAAGGAGGTCCATCGATCACATCAGCATCAGTTTCTTAGTCAAAGTTCCATTTTT

GGCGTATCCAACGGTGGTCATGATGAGTACTTGATCCATCACCATAACGTTAACGATTTC

CGGCAGATGATGTAG

>Chumilis22132.2 +

ATGTCAGAATCCAATCACCAGAACTCAATGTATGGCTCGGGAGGAGCCCCGCAGAGTAAC

CAAGTTGAGGAACAAGAAGACGACGTTGAAGAGTCCATAGACAACCCTCACATACGTTTC

GAAGATAGTAGCGCCATTCCTCCCAATCCACTGTACCTTACCAGCTCAGAATACCCTCCG

GCGGCGGTCACAAACGGCGGCTCTGATCAGCTTACGCTGTCGTTTCAGGGCGAGGTTTAC

GTCTTCGACGAAGTTTCTCCTGATAAGGTGCAGGCCGTACTACTACTTTTGGGCGGATAT

GAAATCCCTTCTGGCATTCCTTCGATGGGGCCGGTACCTCTTAACCAGCAAGGTATGAAT

GACTTACCTGTAAAGCCAATTCAACCACAAAGAGCTGCATCTTTAAGTCGGTTTCGTGAG

AAAAGAAAAGAACGTTGTTTTGATAAGAAAATCCGTTACACTGTGCGAAAAGAAGTTGCA

CTCAGAATGCAGCGTAAGAAGGGTCAGTTTACATCATCCAAGGCTAGTTCTGATGATGGG

GCCCCCGCTTCATCTGGTGCAACACAGGGCTCTGGACAAGATGAGAGCATGCAGGAAACT

TCGTGCATGCACTGTGGGATAAGCTCAAAATCAACTCCAATGATGCGTCGTGGGCCAGCA

GGTCCAAGGACTCTATGCAATGCATGTGGGCTCAAGTGGGCTAACAAGGGAGTTCTAACA

GGTGGTCCTAAGGTTTCAAATATAGGTATGCAGGATCCTTCTGCAAAGGGAATTGAACAG

GGTGATGGCGAAGCTAAAGACTCAGTTGCTATAACCATGGGTGCCAACATCGCCCCTTCC

TCTAATGGTGACAACTCAGCCATGACTGTGGACAGGAATTTATGA

>Chumilis22133.1 +

ATGGAGATGGTGAACGCTCAGCTACTGCAAGCGAGGGCTTACGAGGGGGAAGATCAACTG

GTGCATATACCGGCAGTGTTGGAAGGGGACTATGACGACGGAGGTGGTGCTAAAGCGGCC

ATGAATGGAGGGGACCAAGCTCGGAGGAGCTCTGGAGTAACCATGAGTCGATGCAATTCG

GCTCTGCCTTCTCGGACCAGTGAGCTCACCATCGCCTTCGAGGGCGAGGTCCACGTTTTT

CCCGCGGTTACTCCCGATAAGGTGCAAGCAGTGCTTTTGCTATTGGGAAGGCGTGATATA

TCCAGCAGTTTCCCTAGTTCTGAGTCTCTGCTAGAAAGCAATAGCGGGGGTATAGGTGAC

ATCTCACGAAATTCAAAACTTTCACGAAGAACTGCATCTCTTGTTAGGTTCCGTGAAAAA

CGGAAAGAGAGATGCTTTGAAAAGAAAATTCGATACACTTGTCGAAAAGAGGTTGCTCAG

AGGATGTATCGCAAGAATGGACAGTTTGCATCATTGAAGGACGATTCTAAAATTGCTTCT

GGAAACTGCGATTCAAGTGATGGCACTTCTTGTCCAGAATCTGTTTTACGTAGATGTCAG

CATTGTGGAATTAGTGAAAAGTCTACCCCAGCAATGCGTCGGGGGCCAGCTGGTCCAAGA

TCTCTTTGTAATGCTTGTGGTCTCATGTGGGCTAACAAGGGAACTTTGAGAGATCTTACA

AAACCAGGGAGGCCCATCCATTTTGACCAAAATGAGCTGGAAACTGCAGCTGACTTTAAA

CCTTTGATGTTAAAACCAGAAAATGCGCATCTAGACCGGGATGAAGAGGGAAGCCCAGAG

GAGAGTAAGCCTATTGCATTGGATACTGAAAACCCTCCTCTGAGACTGGGTGACGAGGAC

ATGCCGGAAACAGCTGAAGCTGCTGCTAATCACATATCCATCCAAATGGAGGATTCAACT

GTTAACTTTGACGAGCAGGAGAATCTGGATGAATTTTGCAATGCCTCGGGGACAGAATTT

GAGATTCCTGCAAACTTTGATGAGCAGGTGGTTGATTTTTATGATTGCAACATCGAGACT

CACTGGCCAGGAACTTGA

>Chumilis22257.1 -

ATGGAGCTCTGCATGGAAGCAAAGGCTTTGAAATCAAGTTTACGCAGAGAACTGGCCTTG

AAGTCGAACCAGCAAGCTTTGATTGATGAGTTTTGGTGCGCCACTGGAATTTCTGGTGTA

CCCAGTGAAGATTTTTCAGTGGACGACCTTCTTGACCTCTCTAATGGCGAATTCGAAGAT

GGGTCTGTTGAAGAAGAAGAAGAAGAAGAAAAAGACTCAGTTTCTGTGGATGATGAAAGT

TCTAATTCAAGCAACTTTGTCTCTACCGACTCTGATTCTGCTCTGGCCAGCCAACTCTTA

GTCCCGGATGATGATTTAGCAGGGCTTGAATGGGTTTCTCACTTTGTGGATGATTCTATG

TTAGACCTCTCTCTGCTTCACCCAGTCGGTACCCAAAAACCGGAGGCTCTTGCCTTGACC

CCGTCTGAAACGGAGGCCAAGCCGGTTCAATCAAGACCCACATGGTTTCCTTCGCAAGTT

CCAGTGAAACCCAGGACCAAGCGTTGCAGAGCGGCCAGTAGAGTTTGGTCGTACCCGTCT

TCGTCTTCACCATCGTCGTCGTCTTCTTGTTCTTCTGGGTTTTCATTTTCAACGCCTTGT

CTTACCTTCAACAATCCGGTTCACAGCGCGGACGTGTTTGTCGGTGAACCGGCGACCAAG

AAACAGAAGAAAAAACCGGCGGTTCAAACCGGGGTCGACGGTTCAGTTGGGGTACAGTTT

CAGAGGAGGTGTAGTCATTGCCATGTTCAAAAGACCCCACAATGGAGAACCGGTCCACTT

GGTGCCAAAACTCTATGTAATGCGTGCGGGGTTCGATTCAAGTCTGGTCGGCTTTTTCCC

GAGTATAGACCCGCATGCAGTCCGACTTTTTCTGGTGACGTCCACTCGAATAGTCACCGC

AAAGTGCTGGAGATGAGGAAGAGAAAAGAGGCGGGCGGACCCGAGCCCGGGTTGAACCGA

GTAATTCCGAGTTTTTGA

>Chumilis24079.1 -

ATGGATCAAAGACAGAAAGGATCAGAGGCAGAGCACATGAATCAGAGTAGCATTGCGCCG

CCCAGCAAAAGCTGTACAGATTGCCACACAACAACAACACCATTATGGAGGGGAGGGCCA

GCTGGGCCAAGGACTCTTTGCAATGCATGTGGGATTAAATACAGAAAGAGAAAGAGGGCT

CTTGTTGGTTTAAACAAAGGAGCAACTGGAACAGAGAAAAGGAAACACAAGACTATTGGA

GCCCCTTTGAAGCTGGGATTGAAGTCTATGGAAAGAGAAATGGTCGAAATGGTGTTCCAC

TTCCACAAGAAGAATTTGAAAGAAGAAGAAGAAGCCGCTATACTGTTGATGGCCCTATCT

TGTTCTAGTGGCACTGGCGGTGCCGTCTATGCTTAA

>Chumilis25938.1 +

ATGGGCAAGCAAGGACCTTGCTATCACTGTGGAGTTACAAGCACTCCCCTTTGGCGCAAC

GGGCCACCTGACAAGCCAGTACTTTGCAATGCGTGTGGATCTCGATGGAGGACTAAGGGA

ACACTTGCAAATTATACACCTCTGCATGCTCGAGCCGAACCTGATGATTATGAAGATCAT

AGGGTTTCCAGGGTGAAGAGCATATCTATAAACAAAAACAAAGAAATAAAACTGGTCAAA

AGAAAGCAGAATCCTGACAGTGTGATGGTTGGAGGGGTTGCTGCTGATTATGCCCATGGC

TTCCGAAAGGTAACTGATGAAGATACAAGTAATAGGTCCAGTTCTGGGTCTGCTGTGTCT

AACTCTGAGAGCTGTGCACAATTTGGCAGTGCAGATGCAAGTGATTTGACAGGTCCTGCT

CAGTCAATGGTATGGGACTCAATGGTTCCTTCTAGGAAAAGGACCTGTATCGGTCGTCCT

AAGCCATCTCCTGTTGAAAAGCTTACAAAGGATCTATATACTATTTTACATGAGCAACAG

TCTTCGTACTTTTCCGGGTCTTCTGAAGAGGATCTGCTTTTTGAATGTGAAACACCAATG

GTTTCTGTTGAAATAGGACATGGAAGTGTTCTCATGAGGCATCCCAGCTCAATAACACGA

GAAGAGGAATCTGAAGCTAGCTCCCTTTCAGTTGATAACAAACAGTGCCACATAAATGAG

GCTTATTCCCATCCTGAAACCCTTCTTGTACATAATAATAAGGGTGTCATTATGACCAGT

ACTGTAACTGGAAAGATGAATAACCTTGCAGGACAAGGGATGCAACAAGAGCCACTGAAA

AGGGATAAGTCTCAGTATGACAACTTCCAGATCATGGGAAACCATAATTCACCACTGTGT

CATGTAGATTTGAATGATATTTTAAATTTTGAAGAGTTTACGAGGCAGTTGACAAATGAG

GAACAACAGCAATTACTGAAACATCTACCTCCCGTTGATGTTGTTAAATTTCCTTATAGC

CTCAAAAGCATGTTTGATAGTCCTCAATTCAGGGAGAATTTGACTTCCTTTCAGCAACTG

CTAGCAGAAGGGGTCTTTGATATCTCCTTTCTAGGGGCAAAAACTGAAGACTGTAAGACT

TTGAAAAGGCTTGTATTGTCCAATTCTTCGAAATCTAAATGGGTTGAACGCTATCATCTT

CTCAAGAAATGTAAAACTAGCCCTGGACAGTCTGTTATTTCTGGGCCTAACGCCCTGGCA

TCTAGTAATTTCCGACATGTCAAGAGACTGCGGGACAGCGAGACTCAAAATTTTCCAGAT

GTAAAGATCATGATGAAGAGCCCCAAAAGGATAATCGTGAAGGGTAGCAATGAAAACAAG

GATCTCATGGACTATGATGGTTCTTGCTTTAGTCCAAGAAGCCTATTCGCTTTGCCTGCA

GATGGTAGCTCTCTCTTGATGGAATCTATGAATTTTGTTGATGAAAGTTCTGATCAGGAT

CTTCTACTGGATTTGCCATCCAATGGATCTTTCGCACAAGCAGAGCTTCTTCACCCAGCC

ATGAGTTTTGGTGCCCAACAGACAAGCACTAGTAGTAGCTCAATATACCCACACGTACTC

CATCCGTGA

the information of the proteins used for phylogenetic tree construction

>AtGATA1

MEMESFMDDLLNFSVPEEEEDDDEHTQPPRNITRRKTGLRPTDSFGLFNTDDLGVVEEEDLEWISNKNAFPVIETFVGVLPSEHFPITSLLEREATEVKQLSPVSVLETSSHSSTTTTSNSSGGSNGSTAVATTTTTPTIMSCCVGFKAPAKARSKRRRTGRRDLRVLWTGNEQGGIQKKKTMTVAAAALIMGRKCQHCGAEKTPQWRAGPAGPKTLCNACGVRYKSGRLVPEYRPANSPTFTAELHSNSHRKIVEMRKQYQSGDGDGDRKDCG*

>AtGATA2

MDVYGLSSPDLLRIDDLLDFSNEDIFSASSSGGSTAATSSSSFPPPQNPSFHHHHLPSSADHHSFLHDICVPSDDAAHLEWLSQFVDDSFADFPANPLGGTMTSVKTETSFPGKPRSKRSRAPAPFAGTWSPMPLESEHQQLHSAAKFKPKKEQSGGGGGGGGRHQSSSSETTEGGGMRRCTHCASEKTPQWRTGPLGPKTLCNACGVRFKSGRLVPEYRPASSPTFVLTQHSNSHRKVMELRRQKEVMRQPQQVQLHHHHHPF*

>AtGATA3

MELWTEARALKASLRGESTISLKHHQVIVSEDLSRTSSLPEDFSVECFLDFSEGQKEEEEEVVSVSSSQEQEEQEHDCVFSSQPCIFDQLPSLPDEDVEELEWVSRVVDDCSSPEVSLLLTQTHKTKPSFSRIPVKPRTKRSRNSLTGSRVWPLVSTNHQHAATEQLRKKKQETVLVFQRRCSHCGTNNTPQWRTGPVGPKTLCNACGVRFKSGRLCPEYRPADSPTFSNEIHSNLHRKVLELRKSKELGEETGEASTKSDPVKFGSKW*

>AtGATA4

MDVYGMSSPDLLRIDDLLDFSNDEIFSSSSTVTSSAASSAASSENPFSFPSSTYTSPTLLTDFTHDLCVPSDDAAHLEWLSRFVDDSFSDFPANPLTMTVRPEISFTGKPRSRRSRAPAPSVAGTWAPMSESELCHSVAKPKPKKVYNAESVTADGARRCTHCASEKTPQWRTGPLGPKTLCNACGVRYKSGRLVPEYRPASSPTFVLTQHSNSHRKVMELRRQKEQQESCVRIPPFQPQ*

>AtGATA5

MEQAALKSSVRKEMALKTTSPVYEEFLAVTTAQNGFSVDDFSVDDLLDLSNDDVFADEETDLKAQHEMVRVSSEEPNDDGDALRRSSDFSGCDDFGSLPTSELSLPADDLANLEWLSHFVEDSFTEYSGPNLTGTPTEKPAWLTGDRKHPVTAVTEETCFKSPVPAKARSKRNRNGLKVWSLGSSSSSGPSSSGSTSSSSSGPSSPWFSGAELLEPVVTSERPPFPKKHKKRSAESVFSGELQQLQPQRKCSHCGVQKTPQWRAGPMGAKTLCNACGVRYKSGRLLPEYRPACSPTFSSELHSNHHRKVIEMRRKKEPTSDNETGLNQLVQSPQAVPSF*

>AtGATA6

MESVELTLKNSNMKDKTLTGGAQNGDDFSVDDLLDFSKEEEDDDVLVEDEAELKVQRKRGVSDENTLHRSNDFSTADFHTSGLSVPMDDIAELEWLSNFVDDSSFTPYSAPTNKPVWLTGNRRHLVQPVKEETCFKSQHPAVKTRPKRARTGVRVWSHGSQSLTDSSSSSTTSSSSSPRPSSPLWLASGQFLDEPMTKTQKKKKVWKNAGQTQTQTQTQTRQCGHCGVQKTPQWRAGPLGAKTLCNACGVRYKSGRLLPEYRPACSPTFSSELHSNHHSKVIEMRRKKETSDGAEETGLNQPVQTVQVVSSF*

>AtGATA7

MECVEAFLGDFSVDDLLDLSNADTSLESSSSQRKEDEQEREKFKSFSDQSTRLSPPEDLLSFPGDAPVGDLEDLEWLSNFVEDSFSESYISSDFPVNPVASVEVRRQCVPVKPRSKRRRTNGRIWSMESPSPLLSTAVARRKKRGRQKVDASYGGVVQQQQLRRCCSHCGVQKTPQWRMGPLGAKTLCNACGVRFKSGRLLPEYRPACSPTFTNEIHSNSHRKVLELRLMKVADPARV*

>AtGATA8

MIGTSFPEDLDCGNFFDNMDDLMDFPGGDIDVGFGIGDSDSFPTIWTTHHDTWPAASDPLFSSNTNSDSSPELYVPFEDIVKVERPPSFVEETLVEKKEDSFSTNTDSSSSHSQFRSSSPVSVLESSSSSSQTTNTTSLVLPGKHGRPRTKRPRPPVQDKDRVKDNVCGGDSRLIIRIPKQFLSDHNKMINKKKKKKAKITSSSSSSGIDLEVNGNNVDSYSSEQYPLRKCMHCEVTKTPQWRLGPMGPKTLCNACGVRYKSGRLFPEYRPAASPTFTPALHSNSHKKVAEMRNKRCSDGSYITEENDLQGLIPNNAYIGVD*

>AtGATA9

MEKIAPELFLVAGNPDSFVVDDLLDFSNDDGEVDDGLNTLPDSSTLSTGTLTDSSNSSSLFTDGTGFSDLYIPNDDIAELEWLSNFVEESFAGEDQDKLHLFSGLKNPQTTGSTLTHLIKPEPELDHQFIDIDESNVAVPAKARSKRSRSAASTWASRLLSLADSDETNPKKKQRRVKEQDFAGDMDVDCGESGGGRRCLHCATEKTPQWRTGPMGPKTLCNACGVRYKSGRLVPEYRPASSPTFVMARHSNSHRKVMELRRQKEMRDEHLLSQLRCENLLMDIRSNGEDFLMHNNTNHVAPDFRHLI*

>AtGATA10

MNWLPEAEAEEHLKGILSGDFFDGLTNHLDCPLEDIDSTNGEGDWVARFQDLEPPPLDMFPALPSDLTSCPKGAARVRIPNNMIPALKQSCSSEALSGINSTPHQSSAPPDIKVSYLFQSLTPVSVLENSYGSLSTQNSGSQRLAFPVKGMRSKRRRPTTVRLSYLFPFEPRKSTPGESVTEGYYSSEQHAKKKRKIHLITHTESSTLESSKSDGIVRICTHCETITTPQWRQGPSGPKTLCNACGVRFKSGRLVPEYRPASSPTFIPSVHSNSHRKIIEMRKKDDEFDTSMIRSDIQKVKQGRKKMV*

>AtGATA11

MNWLPEAEAEDDFKGLLSGDFFDDLINHLDVPLDDIDTTNGEGDWVDRFQDLEPPPMDMFPTLPSDLTSCGSGMAKAPRVDIQRNIPALKQSYSSEALSSTLHQSSAPPEIKVSKLFQSLSPVSVLENSYGSLSTHNNGSQRLAFPVKGMRSKRKRPTTLRLSYLFPSEPRKPEKSTPGKPESECYFSSEQHAKKKRKIHLTTRTVSSTLEASNSDGIVRKCTHCETTKTPQWREGPSGPKTLCNACGVRFRSGRLVPEYRPASSPTFIPAVHSNSHRKIIEMRRKDDEQFDSSMIRAVISRG*

>AtGATA12

MEDEAHEFFHTSDFAVDDLLVDFSNDDDEENDVVADSTTTTTITDSSNFSAADLPSFHGDVQDGTSFSGDLCIPSDDLADELEWLSNIVDESLSPEDVHKLELISGFKSRPDPKSDTGSPENPNSSSPIFTTDVSVPAKARSKRSRAAACNWASRGLLKETFYDSPFTGETILSSQQHLSPPTSPPLLMAPLGKKQAVDGGHRRKKDVSSPESGGAEERRCLHCATDKTPQWRTGPMGPKTLCNACGVRYKSGRLVPEYRPAASPTFVLAKHSNSHRKVMELRRQKEMSRAHHEFIHHHHGTDTAMIFDVSSDGDDYLIHHNVGPDFRQLI*

>AtGATA13

MNNDLWLPEEDFKGLPDNFLDNLVDPTNDVSVEDIETGDDEGDWDAKFQKLVPPPLDELMSLSYEFTCNGQRVQVQKHVPILKQSSSSEVFSTVDNSPPNVKVSKLLQSLSPVSVLKNTNGSGSPQNPNGDQKLAFLVKGIRSKRKRPTLLRVTFLKSFLLEMSQQFAPDESESSEISALKKRKKNKSRRLKCTHCETTTTPQWREGPNGRKTLCNACGIRFRSGRLVLEYRPAASPTFIPTVHSNLHKKIIYMRMKDNDQFDTRKIRAETSGPETRSRLRNFGRPMSYGQ*

>AtGATA14

MSGREDEEEDLGTAMQKIPIPVNVFDKEPMDLDTVFGFADGVREIIEDSNLLLEESREFDTNDSKPSRNFSNLPTATRGRLHAPKRSGNKRGRQKRLSFKSPSDLFDSKFGITDKSCSHCGTRKTPLWREGPRGAGTLCNACGMRYRTGRLLPEYRPASSPDFKPNVHSNFHRKVMEIRRERKSSPPNSFGFSESYHSTRKLGF*

>AtGATA15

MLDPTEKVIDSESMESKLTSVDAIEEHSSSSSNEAISNEKKSCAICGTSKTPLWRGGPAGPKSLCNACGIRNRKKRRTLISNRSEDKKKKSHNRNPKFGDSLKQRLMELGREVMMQRSTAENQRRNKLGEEEQAAVLLMALSYASSVYA*

>AtGATA16

MLDHSEKVLLVDSETMKTRAEDMIEQNNTSVNDKKKTCADCGTSKTPLWRGGPVGPKSLCNACGIRNRKKRRGGTEDNKKLKKSSSGGGNRKFGESLKQSLMDLGIRKRSTVEKQRQKLGEEEQAAVLLMALSYGSVYA*

>AtGATA17

MSEGSEDTKTKLDSAGELSDVDNENCSSSGSGGGSSSGDTKRTCVDCGTIRTPLWRGGPAGPKSLCNACGIKSRKKRQAALGMRSEEKKKNRKSNCNNDLNLDHRNAKKYKINIVDDGKIDIDDDPKICNNKRSSSSSSNKGVSKFLDLGFKVPVMKRSAVEKKRLWRKLGEEERAAVLLMALSCSSVYA*

>AtGATA18

MMQTPYTTSTQGQYCHSCGMFHHHSQSCCYNNNNNSNAGSYSMVFSMQNGGVFEQNGEDYHHSSSLVDCTLSLGTPSTRLCEEDEKRRRSTSSGASSCISNFWDLIHTKNNNSKTAPYNNVPSFSANKPSRGCSGGGGGGGGGGGGDSLLARRCANCDTTSTPLWRNGPRGPKSLCNACGIRFKKEERRTTAATGNTVVGAAPVQTDQYGHHNSGYNNYHAATNNNNNNGTPWAHHHSTQRVPCNYPANEIRFMDDYGSGVANNVESDGAHGGVPFLSWRLNVADRASLVHDFTR*

>AtGATA19

MGFSMFFSPENDVSHHSSPYASVDCTLSLGTPSTRLCNEDDERRFSSHTSDTIGWDFLNGSKKGGGGGGHNLLARRCANCDTTSTPLWRNGPRGPKSLCNACGIRFKKEERRASTARNSTSGGGSTAAGVPTLDHQASANYYYNNNNQYASSSPWHHQHNTQRVPYYSPANNEYSYVDDVRVVDHDVTTDPFLSWRLNVADRTGLVHDFTM*

>AtGATA20

MMGYQTNSNFSMFFSSENDDQNHHNYDPYNNFSSSTSVDCTLSLGTPSTRLDDHHRFSSANSNNISGDFYIHGGNAKTSSYKKGGVAHSLPRRCASCDTTSTPLWRNGPKGPKSLCNACGIRFKKEERRATARNLTISGGGSSAAEVPVENSYNGGGNYYSHHHHHYASSSPSWAHQNTQRVPYFSPVPEMEYPYVDNVTASSFMSWN*

>AtGATA21

MDSNFHYSIDLNEDQNHHEQPFFYPLGSSSSLHHHHHHHHHQVPSNSSSSSSSISSLSSYLPFLINSQEDQHVAYNNTYHADHLHLSQPLKAKMFVANGGSSACDHMVPKKETRLKLTIRKKDHEDQPHPLHQNPTKPDSDSDKWLMSPKMRLIKKTITNNKQLIDQTNNNNHKESDHYPLNHKTNFDEDHHEDLNFKNVLTRKTTAATTENRYNTINENGYSNNNGVIRVCSDCNTTKTPLWRSGPRGPKSLCNACGIRQRKARRAAMAAAAAAGDQEVAVAPRVQQLPLKKKLQNKKKRSNGGEKYNHSPPMVAKAKKCKIKEEEEKEMEAETVAGDSEISKSTTSSNSSISSNKFCFDDLTIMLSKSSAYQQVFPQDEKEAAVLLMALSYGMVHG*

>AtGATA22

MGSNFHYTIDLNEDQNHQPFFASLGSSLHHHLQQQQQQQQHFHHQASSNPSSLMSPSLSYFPFLINSRQDQVYVGYNNNTFHDVLDTHISQPLETKNFVSDGGSSSSDQMVPKKETRLKLTIKKKDNHQDQTDLPQSPIKDMTGTNSLKWISSKVRLMKKKKAIITTSDSSKQHTNNDQSSNLSNSERQNGYNNDCVIRICSDCNTTKTPLWRSGPRGPKSLCNACGIRQRKARRAAMATATATAVSGVSPPVMKKKMQNKNKISNGVYKILSPLPLKVNTCKRMITLEETALAEDLETQSNSTMLSSSDNIYFDDLALLLSKSSAYQQVFPQDEKEAAILLMALSHGMVHG*

>AtGATA23

MDPRKLLSCSSSYVSVRMKEEKGTIRCCSECKTTKTPMWRGGPTGPKSLCNACGIRHRKQRRSELLGIHIIRSHKSLASKKINLLSSSHGGVAVKKRRSLKEEEQAALCLLLLSCSSVLA*

>AtGATA24

MDDLHGRNGRMHIGVAQNPMHVQYEDHGLHHIDNENSMMDDHADGGMDEGVETDIPSHPGNSADNRGEVVDRGIENGDQLTLSFQGQVYVFDRVSPEKVQAVLLLLGGREVPHTLPTTLGSPHQNNRGLSGTPQRLSVPQRLASLLRFREKRKGRNFDKTIRYTVRKEVALRMQRKKGQFTSAKSSNDDSGSTGSDWGSNQSWAVEGTETQKPEVLCRHCGTSEKSTPMMRRGPDGPRTLCNACGLMWANKGTLRDLSKVPPPQTPQHLSLNKNEDANLEADQMMEVTGDISNTQ*

>AtGATA25

MFGRHSIIPNNQIGTASASAGEDHVSASATSGHIPYDDMEEIPHPDSIYGAASDLIPDGSQLVAHRSDGSELLVSRPPEGANQLTISFRGQVYVFDAVGADKVDAVLSLLGGSTELAPGPQVMELAQQQNHMPVVEYQSRCSLPQRAQSLDRFRKKRNARCFEKKVRYGVRQEVALRMARNKGQFTSSKMTDGAYNSGTDQDSAQDDAHPEISCTHCGISSKCTPMMRRGPSGPRTLCNACGLFWANRGTLRDLSKKTEENQLALMKPDDGGSVADAANNLNTEAASVEEHTSMVSLANGDNSNLLGDH*

>AtGATA26

MGKQGPCYHCGVTNTPLWRNGPPEKPVLCNACGSRWRTKGTLVNYTPLHARADGDENDDHHRFQRMKSISLGNKNKEIKMLKRKAIQENIIIKRPVFEFSYGLKAAVIEEDASNRSSSGSAVSNSESCAQFSSADGSPSQSNAWDTTVPCKRRTCVGRPKSSSVEKLTKDLYNILQEQQSSCLSVSSEEDLLFENEMSMVSVEIGHGSVLMKNPHSFAREEESEASSLSSIENKSSISDAYSHSVKRVEIGAVRGSYYGGQTIKQEQFKRTKSQTERVHVLGSHGSPLCSIDLKDVFNFDEFIEQFTEEEQKKLMNLLPQIDSDDLPHSLRMMFESAQFKDNFSLFQQLIADGVFDVSSSSGAKLEEIRTFKKLALTDFNKSRLVESYNLLKEREKGTGDSVTTTSKSSIPNVPKNIVTIKRRYENQIQVKSESRGLMRSPKRVMKMKASHETENNVSCFRPRSLASVFAQEGGSAVFSYEGNCSSDQDLLLLDLPSNGSFPQAELLHQL*

>AtGATA27

MGKQGPCYHCGVTSTPLWRNGPPEKPVLCNACGSRWRTKGSLVNYTPLHARAEGDETEIEDHRTQTVMIKGMSLNKKIPKRKPYQENFTVKRANLEFHTGFKRKALDEEASNRSSSGSVVSNSESCAQSNAWDSTFPCKRRTCVGRPKAASSVEKLTKDLYTILQEQQSSCLSGTSEEDLLFENETPMLLGHGSVLMRDPHSGAREEESEASSLLVESSKSSSVHSVKFGGKAMKQEQVKRSKSQVLGRHSSLLCSIDLKDVFNFDEFIENFTEEEQQKLMKLLPQVDSVDRPDSLRSMFESSQFKENLSLFQQLVADGVFETNSSYAKLEDIKTLAKLALSDPNKSHLLESYYMLKRREIEDCVTTTSRVSSLSPSNNNSLVTIERPCESLNQNFSETRGVMRSPKEVMKIRSKHTEENLENSVSSFKPVSCGGPLVFSYEDNDISDQDLLLDVPSNGSFPQAELLNMI*

>AtGATA28

MDDLHGSNARMHIREAQDPMHVQFEHHALHHIHNGSGMVDDQADDGNAGGMSEGVETDIPSHPGNVTDNRGEVVDRGSEQGDQLTLSFQGQVYVFDSVLPEKVQAVLLLLGGRELPQAAPPGLGSPHQNNRVSSLPGTPQRFSIPQRLASLVRFREKRKGRNFDKKIRYTVRKEVALRMQRNKGQFTSAKSNNDEAASAGSSWGSNQTWAIESSEAQHQEISCRHCGIGEKSTPMMRRGPAGPRTLCNACGLMWANKGAFRDLSKASPQTAQNLPLNKNEDANLETDHQIMITVANDISNSQ*

>AtGATA29

MEPELDLTLKLGLPNSTVETHLTLSPPTTTTDQGTNVVDGGEVINHRRGLLGDDEVIHNEPTRNNVEFNIRIYNYVFQQFVGAPNTLNFAPYPMPPSPAPAPETPPVSDEYVLIDVPARRARRNNSTVMTNSWKENATPKRIRGCGGFCGGRIEGMKKCTNMNCNALNTPMWRRGPLGPKSLCNACGIKFRKEEERKAKRNVVIVLDD*

>AtGATA30

MSMTEETKTTKLESAGDSSDVDNGNCSSSGSGGDTKKTCVDCGTSRTPLWRGGPAGPKSLCNACGIKSRKKRQAALGIRQDDIKIKSKSNNNLGLESRNVKTGKGEPVNVKIAKCEPGIVKIAKGEPGNVKNKIKRDPENSSSSNNNKKNVKRVGRFLDFGFKVPAMKRSAVEKKRLWRKLGEEERAAVLLMALSCG*

>OsGATA1

MEVTAEFGGAYYGGAAGREKKALQQGCGDHFAVDDLLVLPYGEEDETTREGEATGGKEEAAGFGNASADSSTITALDSCSNSFGLADGDFPGELCEPYDQLAELEWLSNYMNEGDDAFATEDLQKLQLISGIPSGGFSTASVPSAQAQAASAAASMAVQPGGFLPEAPVPAKARSKRSRAAPGNWSSRLLVLPPPPASPPSPASMAISPAESGVSAHAFPIKKPSKPAKKKDAPAPPAQAQLSSVPVHSGGSAPAAAAGEGRRCLHCETDKTPQWRTGPMGPKTLCNACGVRYKSGRLVPEYRPAASPTFMVSKHSNSHRKVLELRRQKEMHQQTPHHHQPQVAAAGGVGSLMHMQSSMLFDGVSPVVSGDDFLIHHHLRTDFRPPI*

>OsGATA2a

MTHQALIPSTPPSAFSPASHFLHASSSSSPSLSSHAVVATAAAAMSSFAHHHHGSLVEKDGRMSALRSSLRPYEAAEEMAAAAAAGGPAAAWGAVERGAGMMGDGFSVEDLLDLEELCEVDRDGGEQGEAAAAAAAAVEKERSSDSHGSSVVSYEPMPLLPPVMDLPAHDVEELEWVSRIMDDSLAELPLPQLPAAAAALAACGKPQHRRPHEGAASALLDPMRTPTICALSTEALVPVKSRRSKRSRASVWSLSGAPLSDSTSSSSTATTSSCSSSASFSPFLQYVDFPALVASDLLDEQPRSKKSKHGKNGKQKPKKRGRKPKHQQPPHLAAAAGGGAALPATGDRRCSHCGVQKTPQWRAGPEGAKTLCNACGVRYKSGRLLPEYRPACSPTFVSSLHSNSHRKVLEMRRKKETPVIVAAAAPAVASF*

>OsGATA2b

MTHQALIPSTPPSAFSPASHFLHASSSSSPSLSSHAVVATAAAAMSSFAHHHHGSLVEKDGRMSALRSSLRPYEAAEEMAAAAAAGGPAAAWGAVERGAGMMGDGFSVEDLLDLEELCEVDRDGGEQGEAAAAAAAAVEKERSSDSHGSSVVSYEPMPLLPPVMDLPAHDVEELEWVSRIMDDSLAELPLPQLPAAAAALAACGKPQHRRPHEGAASALLDPMRTPTICALSTEALVPVKSRRSKRSRASVWSLSGAPLSDSTSSSSTATTSSCSSSASFSPFLQYVDFPALVASDLLDEQPRSKKSKHGKNGKQKPKKRGRKPKHQQPPHLAAAAGGGAALPATGDRRCSHCGVQKTPQWRAGPEGAKTLCNACGVRYKSGRLLPEYRPACSPTFVSSLHSNSHRKKETPVIVAAAAPAVASF*

>OsGATA3

MAGVGFVEDMLREQSLLEATCGDLFDHIDDLLDFPKEESAADVLLLDAPAPGSPLSSRIIGGHATMAAAPPPPPQMMALPPPPAPAKDDASALFDAAGALGAEVFDRKDAHIGPCDELDMDMAQLEWLSGLFDDGTIPHEPSFPGVNCAAPIKASALTANAGVVLPDKAEEALFRSSSPISVLEHSGFNVATNGGSSSSSSSSASSSSESFSGSGRAWSAPVSPRPEPPVLVIPARARSKRSRPSAFPAVRGAPAATETTILVPTPMYSSTSSHSDPESIAESNPHPPPMKKKKKAKKPAAPAAASDAEADADAADADYEEGGALALPPGTVRRCTHCQIEKTPQWRAGPLGPKTLCNACGVRYKSGRLFPEYRPAASPTFMPSIHSNSHKKVVEMRQKATRTADPSCDLLQYIRRRD*

>OsGATA4

MVGDKDAAALAGELTGDAGASLNGFFDHTGLESAVVGEGQGEGEEEEELEWLSNKDAFPSVDTMAAEVESAAPGAPARAAVGPRTKGLRRRRRVTAPWSLAPLLSRPRQAAAAAADAGAPRRRCTHCAVDETPQWRLGPDGPRTLCNACGVRFKSGRLFPEYRPANSPTFSPLLHSNSHRRVMEMRLQSEEDASAASRVNAKARRAERAAARLAGKDKK*

>OsGATA5

MDALKSSCRSEEAADEGAAAAPSAWGMVERDGFSVEDLLDLEEFCEAEKDAAEENEQALALVAAPEEEKSKDDSQPSSVVTYELVAPPPPPPEIVDLPAHDVEELEWVSRIMDDSLSELPPPPQPPASVVASLAARPPQPRQLQRRPQDGAYRALPPASYPVRTPTICALSTEALVPVKAKRSKRSRATAWSLSGAPPFSDSTSSSSTTTTSSCSSSASFSSFSPLLKFEWHPLGGTSDLPDDHLLPPGKKSKHGKNGKNKPKKRGRKPKQLPPHPSGAAASAPAPGDRRCSHCGVQKTPQWRAGPEGAKTLCNACGVRYKSGRLLPEYRPACSPTFVSAIHSNSHRKVLEMRRKKEVGSGLLTAAAAAAPAVASF*

>OsGATA6

MEVAAADYAGGVRVKKEAGGCGGSGDMFLVDDLLDLPCDEEEEETGLCGAYGGGGAGLGAGVVGGGGDDRAAGNASADSSTVTAVDSCSNSFSGLADGDFSGGLCEPYEQLAELEWVSTYMGEETLPTEDLRKLQLISGIPAAPRAPPALAVSAVQLPAGGAGALPTEAPVPGKARSKRSRVAPCSWSSRLMVLPPPPASPPSPASAVISPSESGTAAPAFPAKKAAKSAKKKDGPSPAPAPNAAAQAAAEGRRCLHCETDKTPQWRTGPMGPKTLCNACGVRYKSGRLVPEYRPAASPTFVVSKHSNSHRKVVELRRQKEMQLLHHHQQPPPHVGAGGGGAAGGLLHVTSPLLFDGPTSSAPLFAGADEFLIHNRISPDYRRQAT*

>OsGATA7

MASEWEMAMGVDLGMGMSTYHNASGGIAAAPMMGHHGGGGGGGGYSAAHHHHHHYYGMPHQAAMGDAMRVDDLLDLSNTPGAHDFFPASAAAAAAGDHGHHHHHHIGGMGEPSGATPSATSSDHQTSMLSFADDFYIPTEDAAELEWLSKFVDDSYSDMPNYQSSAHAAMAAAAASAANNGGGSSAGQDSCLTAAPGRGARSKRSRATAAAAAAWHSLVPRPPSQSSPSSSCSSSDFPSSNKPSGTARPNGSGGGSRGKKSPGPAGAEVGMEAGVRRCTHCASEKTPQWRTGPLGPKTLCNACGVRFKSGRLMPEYRPAASPTFVLTQHSNSHRKVMELRRQKELLIIRGSHRDAAAAAAAAAAAAAAGSAAATGRPELMFRDYGVC*

>OsGATA8a

MGSTDRKVVGIGVAEEGRRSCVECRATTTPMWRSGPTGPRSLCNACGIRYRKKRRQDLGLDLNQPQKQEHGEVIPEVKDSNSNSNNCNSGSGNSSSNLQVVPKRRLLMGVEEAALLLMTLSSPSASTLLHG*

>OsGATA8b

MWRSGPTGPRSLCNACGIRYRKKRRQDLGLDLNQPQKQEHGEVIPEVKDSNSNSNNCNSGSGNSSSNLQVVPKRRLLMGVEEAALLLMTLSSPSASTLLHG*

>OsGATA9

MLHEAAPCTCGLLYGSCGGGCSLLFPAGAPGDHHHHHHYKQYCGAGDGEYPDVPYGGGGSVDCTLSLGTPSTRRAEAAVAGLPWDQSSLQPSCNGRQEMSGAAAPRTEPSGGAGAAAASAPRRCANCDTTSTPLWRNGPRGPKSLCNACGIRYKKEERRAAAAAVAPTALASDGGVEYAYGYPRQQQQWGCYGPAVAKAASFGMFGDAAGEDGPCLPWGLGVMPSSPAFGAVREMPSLFQYY*

>OsGATA10

MDMDSSSSPVDKVDPDECNGSKACADCHTTKTPLWRGGPGGPKSLCNACGIRYRKRRRAALGLDSSATATATDGAEQQKKTKAKKEKAQEEEVTMELHTVGFRSKDAAVFKQRRRMRRRKCLGEEERAAILLMALSSGVIYA*

>OsGATA11

MSTIYMSQLPATLPLMEGDQDQGLYPAFHRAKDPPILFPFMIDSAVEHQGQIYGDQGLRRQQVLGESNQQFNDHMMMGGSDVFLTPSPFRPTIQSIGSDMIQRSSYDPYDIESNNKQHANGSTSKWMSTPPMKMRIIRKGAATDPEGGAVRKPRRRAQAHQDESQQQLQQALGVVRVCSDCNTTKTPLWRSGPCGPKSLCNACGIRQRKARRAMAAAANGGAAVAPAKSVAAAPVNNKPAAKKEKRAADVDRSLPFKKRCKMVDHVAAAVAATKPTAAGEVVAAAPKDQDHVIVVGGENAAATSMPAQNPISKAAATAAAAAASPAFFHGLPRDEITDAAMLLMTLSCGLVHS*

>OsGATA12

MDSSSVEKGSGSIDPDERTASGEPKACTDCHTTKTPLWRGGPSGPKSLCNACGIRYRKKRREALGLDAGEGGAERQEKKKSKRERGEEVTMELRMVGFGKEVVLKQRRRMRRRRRLGEEEKAAILLMALSSGVIYA*

>OsGATA13

MGSSDQKVIGIAAAAAAAAEEAGRRCCVECGATTTPMWRGGPTGPRSLCNACGIRYRKKRRQELGLDKKQQQEHHPHHHQQQQQQYQRQQQQQQQEDHSDAASSVKDSSSSSSNKSSSLQVISEIVISRSKSDCEGAMEGNCVPLKRLVQQVDFLLSSTGITESCQCVAVSCANQMGLQKAANVLLFLVPIRVLTMENNSCDILHIIRIIGRGCGIESKTRIIDR*

>OsGATA14

MLQELAPCTCGMLYGSCGGGCGGAAAAASAFSLLFPMAGGQYYYRQCGGVAEEDSRSPYGGGGAAVDCTLSLGTPSTRRAEAGAYGGGLQPWDVPSSARPGGGGGGKQDGAGVAPCNKEAPAAGRLPRRCANCDTMSTPLWRNGPRGPKSLCNACGIRYKKEERRAAAAVAPTPPPSLDTGAGYAYCYSRQPPPPPAPQWGCYGQAAAKSASYAMFDAADDGPCLSWRLNMMPSSPAFAVGERPGLFQYY*

>OsGATA15

MLHHYYSGGAGHHQDVAAAGSPGDMASSTFSLFFPMSNGQCWPPSTVEESAAYDDHSTVTTSPSSPSSSSTGSVDCTLSLGTPSSRRAEPVAAAAPAANHGAPVPAHYPSLSAATVSWDATAESYYCGQQGRPATGAAKCAAGAGHDALLDRRCANCGTASTPLWRNGPRGPKSLCNACGIRYKKEERRAAATTTTADGAAGCGFITAQRGRGSTAAKAAPAVTTCGEETSPYVVGGGGGGGEVADAAYLAWRLNVVPPAATATAFSVWPERASLYHYN*

>OsGATA16

MSTIYMSQLSAALPLMEGEHHHHHQDHHQGHFQAFSLQPKDPPVLFPFVISRRSSSSSPSDSTTLSYGSDHHLTQQQQHQHQAMLEPQNMIGGSSAGIFATPFPTVKSIRDDMIERSQFDPYDTEKLQASCGLAKVVAGGKWSAVPAAKMKITRKMGEPSSGVTGGAATTVAPKKPRRRPAQAYEDHGHGGAMGQAFGVIRVCSDCNTTKTPLWRSGPCGPKSLCNACGIRQRKARRAMMASGLPASPNAAGPKAAAHSGAAAVAAAQPKVKKEKRADVDRSSLPFKKRCKVVQVEDHQTLPAATNAAAAAAMEETAESATVAPPPAPTTRGGTLVDSIGLSWSKTHAAATASCSFRPSPVAPGFAAAVQDEITDAAMLLMTLSCGLVRS*

>OsGATA17a

MSGHHEAKPYQPRRGPAPADEEAAPAAAADEAEAEAEVEAMERYEQEQEYEEGEEGEEEEYEGGEGVPMDADASAAAVAGMDPHGEMVPVAGGEAGGGYPHVASNTLTLSFQGEVYVFESVSAERVQAVLLLLGGRELAPGSGSVPSSSAAYSKKMNFPHRMASLMRFREKRKERNFDKKIRYTVRKEVALRMQRNRGQFTSSKSKAEEATSVITSSEGSPNWGAVEGRPPSAAECHHCGISAASTPMMRRGPDGPRTLCNACGLMWANKGTMREVTKGPPVPLQIVPAATNDVQNGIVEATGVEQHNSAVEEAVSAANGHESQSGVA*

>OsGATA17b

MSGHHEAKPYQPRRGPAPADEEAAPAAAADEAEAEAEVEAMERYEQEQEYEEGEEGEEEEYEGGEGVPMDADASAAAVAGMDPHGEMVPVAGGEAGGGYPHVASNTLTLSFQGEVYVFESVSAERVQAVLLLLGGRELAPGSGSVPSSSAAYSKKMNFPHRMASLMRFREKRKERNFDKKIRYTVRKEVALRMQRNRGQFTSSKSKAEEATSVITSSEGSPNWGAVEGRPPSAAECHHCGISAASTPMMRRGPDGPRTLCNACGLMWANKQNGIVEATGVEQHNSAVEEAVSAANGHESQSGVA*

>OsGATA18a

MPDAAAAAAAAQDADAVMRDAPADAAAGGGDNDDDDGDDGTEEDEEEDDDEEGDEEELPPAEDPAAPEPVSALLPGSPNQLTLLFQGEVYVFESVTPEKVQAVLLLLGRSEMPPGLANMVLPNQRENRGYDDLLQRTDIPAKRVASLIRFREKRKERNFDKKIRYAVRKEVALRMQRRKGQFAGRANMEGESLSPGCELASQGSGQDFLSRESKCQNCGTSEKMTPAMRRGPAGPRTLCNACGLMWANKGTLRNCPKAKVESSVVATEQSNAAVSPSGIDNKELVVPNPENITASHGEVMGDSTPANEAEIGAPKAQSQ*

>OsGATA18b

MPDAAAAAAAAQDADAVMRDAPADAAAGGGDNDDDDGDDGTEEDEEEDDDEEGDEEELPPAEDPAAPEPVSALLPGSPNQLTLLFQGEVYVFESVTPEKVQAVLLLLGRSEMPPGLANMVLPNQRENRGYDDLLQRTDIPAKRVASLIRFREKRKERNFDKKIRYAVRKEVALRMQRRKGQFAGRANMEGESLSPGCELASQGSGQDFLSRESKCQNCGTSEKMTPAMRRGPAGPRTLCNACGLMWANKVLLHVLMS*

>OsGATA19a

MAAEPPADGRDPPADDGAAGDGAVESAAAEALLSAASEQLTLVYQGEVYVFDPVPPQKVQAVLLVLGGSDMPPGLVSMAVPTTFDEKSTTVAARRVASLMRFREKRKERCFDKKIRYSVRKEVAQKMKRRKGQFAGRADFGDGSCSSAPCGSTANGEDDHIRETHCQNCGISSRLTPAMRRGPAGPRSLCNACGLMWANKGTLRSPLNAPKMTVQHPADLSKTGDTDDSKANLCAEHNQTTMKTDTEMVPEQEQKADVLPPTKEEDSMATS*

>OsGATA19b

MTDCYVSVIQVQAVLLVLGGSDMPPGLVSMAVPTTFDEKSTTVAARRVASLMRFREKRKERCFDKKIRYSVRKEVAQKMKRRKGQFAGRADFGDGSCSSAPCGSTANGEDDHIRETHCQNCGISSRLTPAMRRGPAGPRSLCNACGLMWANKGTLRSPLNAPKMTVQHPADLSKTGDTDDSKANLCAEHNQTTMKTDTEMVPEQEQKADVLPPTKEEDSMATS*

>OsGATA20

MARFEEEHRALGAEEEYEEEEDELEEEEEEMEEDEDAQHHEGVGGEVAVPMDAEAAAQLDPHGGMLAASGAVQPMASNQLTLSFQGEVYVFDSVSPDKVQAVLLLLGGRELNPGLGSGASSSAPYSKRLNFPHRVASLMRFREKRKERNFDKKIRYSVRKEVALRMQRNRGQFTSSKPKGDEATSELTASDGSPNWGSVEGRPPSAAECHHCGINAKATPMMRRGPDGPRTLCNACGLMWANKGMLRDLSKAPPTPIQVVASVNDGNGSAAAPTTEQEIPAPATVNGHESST*

>OsGATA21a

MGKQGPCRHCGVTSTPLWRNGPPDKPVLCNACGSRWRTKGSLTNYTPMHARDDIDAEEPRASKLKPPTLKLKEQKQLKKNPSHITMENGPFSDQNFRKMGDPDLSNRSGSGSALSYSESCAPYGTADASEMTASAQSHAWESLVPSKRRSCVTRPKPSQMEKLAKDLNSIMHEEQLLYLSGSSEEDLIYHSATPVDSFEMGYGSMLLRPNSKSLEEESEASSIPADNKSYITSESYSGSVSFVYSESKATSNQNVITEQPKKFLVQTSDNARRANLHTENQDTLENANSPLVSLHMEGKDSEETRVKTSASNRLTKSTMNPLKRPHDTHFQSSVELRGTMRSPKRVSKYGDAMGLKCQASFMPKPGNGKDLACSDRALNLFMLPPDKLSMLVPPQYANTDSDQDLLLDVPLNARHPEAELLCQPSQLSSVAHSSTSEAGNAGGEGRLKQP*

>OsGATA21b

MGKQGPCRHCGVTSTPLWRNGPPDKPVLCNACGSRWRTKGSLTNYTPMHARDDIDAEEPRASKLKPPTLKLKEQKQLKKNPSHITMENGPFSDQNFRKMGDPDLSNRSGSGSALSYSESCAPYGTADASEMTASAQSHAWESLVPSKRRSCVTRPKPSQMEKLAKDLNSIMHEEQLLYLSGSSEEDLIYHSATPVDSFEMGYGSMLLRPNSKSLEEESEASSIPADNKSYITSESYSGSVSFVYSESKATSNQNVITEQPKKFLVQTSDNARRANLHTENQDTLENANSPLVSLHMEGKDSEETRVKTSASNRLTKSTMNPLKRPHDTHFQSSVGQQLLTCIYCYLYLEKKQILYIHFLFRP*

>OsGATA22

MQFEDGGDVHVGAGEGEDGGRVTVDELTRCLRCGISANATPHMRRGPEGRRTLCNACGIAWAKGKVRKVIDSDTPMDNAMFAQMVPELSMEFDDEDKAYEFYNRYAGHVGFSVRKSSSDKSAENITRSRTFVCSREGFRKDKKGAKEVKRPRPETRIGCPARMSIKITSDGKYRISEFVPDHNHQPAPPSTMHMLRSQRVLTELQTTEADSSEESATPSRFSSCSLVKQAEVIRHTNFLPAEYRCSLCSKRKKNMQPGDAGVTVKYLQSMQLSNPSFFYAVQLDEDDKLTNIFWADSKSRTDFSYYSDVVCLDTTYKINEHSRPLTLFLGVNHHKQISIFGAALLYDESEESFKWLFDTFKIAANGKQPKTILTDWSMAATTASAITAAWPGTVHRLCPWQVYQNSVKHLNHIFQGSKTFAKDFGKCVYDYDDEENFLLGWNTMLEKYDLRNNEWIKKIFDDRDKWSPVYNRHVFTADIKSSLQSESVRNALKKSLSPQFDLLSFFKHYERMLDEFRYAELQADFHASQSFPRIPPSKMLRQAANMYTPVVFEIFRREFEMFVDSVIYSCGEDGNAFEYRVAVTDRPGEHYVRFDSGDLSVVCSCKKFEAMGIQCCHVLKVLDFRNIKELPQKYFMKRWKKDVKSASTGNQELLNGGVSQIPSSYLNVPVPFIDPQHVQSNNELNHDTSVSNSHQQALHGGAQGSQGYAPLAGIQQQQFIGNFRLNHETGFL*

>OsGATA23a

MRVWLGRVGGGDAMMHMLVAPDGGGGGEMPPPYGGAAAAPPPPMEQELELHRDNADDGLDGHVRCLRCGISGNATPHMRRGPDGPRTLCNACGIAYRKGKMRRMIEAEPPIDEAALAKLVPEVGMEFESEDKAYEFYNKYAGHVGFSVRKSTSHKSSGNITKVRTFVCSREGYNRDKKSLEAKKPRLDTRIGCPARLIIKVTPESKYRVTDFKADHNHQLAPPSTMHMLRSQRILTELQSGEAELSDDSVMTPTTKATGDLVVRQIGFLRSISLLPADYKNYLRSKRMKAMQLGDGGAILKYLQTMQMENPAFFYTMQIDEDDKLTNFFWADPKSREDFNYFGDVLCLDTTYKINGYGRPLSLFLGVNHHKQTIVFGAAMLYDESFESYRWLFESFKIAMHGKQPAVALVDQSIPLASAMAAAWPNTTQRTCAWHVYQNSLKHLNHVFQGSKTFAKDFSRCVFGYEEEEEFLFAWRSMLEKYDLRHNEWLSKLFDERERWALAYERHIFCADIISALQAESFSSVLKKFLGPQLDLLSFFKHYERAVDEHRYAELQADFQASQSYPRIPPAKMLKQAAHTYTPVVFEIFRKEFELFMDSVLFSCGEAGATSEYKVAPSEKPKEHFVRFDSSDCSCICTCRKFEFMGIPCCHMLKVLDYRNIKELPQRYLLKRWRRTAKSANEENQGTLLFLICMRIHFAEAHDAGDLNLNIIPSARCLLHDTGVEAVRTGHAELPRIKVSNL*

>OsGATA23b

MRVWLGRVGGGDAMMHMLVAPDGGGGGEMPPPYGGAAAAPPPPMEQELELHRDNADDGLDGHVRCLRCGISGNATPHMRRGPDGPRTLCNACGIAYRKGKMRRMIEAEPPIDEAALAKLVPEVGMEFESEDKAYEFYNKYAGHVGFSVRKSTSHKSSGNITKVRTFVCSREGYNRDKKSLEAKKPRLDTRIGCPARLIIKVTPESKYRVTDFKADHNHQLAPPSTMHMLRSQRILTELQSGEAELSDDSVMTPTTKATGDLVVRQIGFLRSISLLPADYKNYLRSKRMKAMQLGDGGAILKYLQTMQMENPAFFYTMQIDEDDKLTNFFWADPKSREDFNYFGDVLCLDTTYKINGYGRPLSLFLGVNHHKQTIVFGAAMLYDESFESYRWLFESFKIAMHGKQPAVALVDQSIPLASAMAAAWPNTTQRTCAWHVYQNSLKHLNHVFQGSKTFAKDFSRCVFGYEEEEEFLFAWRSMLEKYDLRHNEWLSKLFDERERWALAYERHIFCADIISALQAESFSSVLKKFLGPQLDLLSFFKHYERAVDEHRYAELQADFQASQSYPRIPPAKMLKQAAHTYTPVVFEIFRKEFELFMDSVLFSCGEAGATSEYKVAPSEKPKEHFVRFDSSDCSCICTCRKFEFMGIPCCHMLKVLDYRNIKELPQRYLLKRWRRTAKSANEENQGYVANGNGSSLNSIVPPANHHGLQGFSAMIQDTPVSNMHENSFRRSS*

>OsGATA24

MVVVDGLHDGGGGDLQALLDDAGVDDVAARGGGEVEEEVERPSNEDAFPAVEKMATAAAAKGLQCRHCGTTETPQWRHGPEGHRTLCNACSMRYRSGKLVPEYRPLRSPTFSPELHSNRHHRVLQLRRRPGPQSAAPSPAAVARYGGEAKEEEEELAWVSNKDAFATVETTMAPSPRVVETPPEHDHRPANTPTTSPEPHSDRPRRVVQLPRRLQEPSASANLAHAVAATARAGRECAHCGTTKTPAWRLGPDSRRKLCNACGNKYRSGQLNSTTFSQNSQEQKKKSKSSACSRERKRSAVAATVVVGGGLRDDAAAIADEHLDGGDLQALLDDVALDDVAARGGGDAGEAKEEEEELEWLSNKDAFPTVETMSPAPPENRTKAPVPPAGWQCRHCGSTETPLWRERDGPAEAEHVRKEETPPNITPATKHRRIVDLLRCSTALNTAAKAVERRCTHCGTTKTPAWLSGPDSRGKLCNACGKQYRKGRLVPEYRPLNCPTFSPELHSNAHAHRRRRESPVAIAIAGEK*

>OsGATA25

MDVHPPNAAASSLEELFPHQPATESDRSGIEWLSVYVEDCLSTSASCTNPVSAELPPITMASQGAAKPKLPPRSSTNARKKKRSLASVISDTDDQHCITLFVEPPLLLLDHKDWLAESELILPKKDKDEELVQEQEQEEEENYKMSAGMQFQQEQLVITCSYCLSSQSPQWWDGPSGPTCDACRLRIEARNGHTTSSKKRYGQEIDKEQDIGKRRDKKKIKKAVYVNDELLSEEPMKRCTHCLSYKTPQWRTGPLGPKTLCNACGVRFKSGRLLPEYRPANSPTFVSDIHSNSHKKVMQLRNSVPHPRK*

>OsGATA26

MDGDGDVGGGGGGGGGGGVRYVLALPAMASLAVLIAHLDAAVPVPRRPRSYLPRAVPMAWWAFRLPVFRPPPPPPPPPAKNPVKEEEGVARVVVVVAPPPPVDPGEEEAGKRAAKRARRCLNCDAVETPQWRSGPMGRSTLCNACGVRLRAVGSLPEHRAPAARTTTAAPASPPDSPIWTPGHKPPSSSPDIYLVRRTPKLPVTRPPRTKQAPPTAPAPAPPPPPPQPASPKTKTKAKAKKPKRKRSCVHCGSTETPQWREGPTGRGTLCNACGVRYRQGRLLPEYRPKGSPTFSPSVHAANHRQVLELRRQQRQSTNPSTPPPPPVSAAEPIPDEQKEEVVSVPVAAAAPATDGGAASSLDALLLDGPSAPLIVDGDDFLVS*

>OsGATA27

MRKPTPYVSLHDVVAFDFVDGDVPFDDLVDGEGLCCPDDPFEEVMRCLSAVDDPFLAAFKLDCSPPTPAADADVDSRSEEHMHADVGGGLDLQRAVGGGDEKAGTPSTVDDVPWLQASAVARKPRRAPAAVRKRVWSLVSPQLATAAAAAVDNSRDEVSSGGGGGGEGGEHCSRPAKRRRKCGEEKRCGHCQTTETPQWRVGPDGPSTLCNACGIRYRIDHLLPEYRPSTSPGFGSDGYSNRHRKVVKLREKKRKKAMLAATATALTSGPV*

>OsGATA28

MPKPTPSSSSFLDFTGGVDGDDDDPSCPFEGLCCPDDPLDQVLNFDSSDFGHVFFESLDVELFLPRGGPSRGAGEEDSKGAVERVAFGSSAAVESELGGVGGGGAGSEVSVPGGAGGGRGEDMETEALDVKPVVGVGAGGAMGAHVAGGVGAPGAFPESKQLVPWPCAVGAGASAPGAAPDNRLLALPDVRFDALTAEGAAPGGERGKTIPDSVSKNGLPTLPGVRSATPTAPPATPFRLEWDHAAAPSSSATTTPSDSSLSSPPSLSSVFPRIARVFPSRTKPRRRRTLRRQHWSLICPLHLVPVAAAADAARGKSISELNASASAATDAGTPSINDGGGGSYHRRVVGRQRNRQVRKDRRCSHCGTSETPQWRMGPDGPGTLCNACGIRSKMDRLLPEYRPSTSPSFNGDEHSNRHRKVLKLREKKGRD*

>ChGATA01

MAAVNPQPLQARPFEEHGRGPIPIEDDEAEYEDGGDDGMEDTEEVHVNSVSVAERGGGGGGGGGGGVVMASRTSELTLSFEGEVYVFPAVTPEKVQAVLLLLGGRDVPTGVPTVEVSYDQNTRGVADTPKRSNLSRRIASLVRFREKRKERCFDKKIRYTVRKEVAQRMLRKNGQFASLKQNSGDSGWDSAQSGLQDGTSRPETVLRRCQHCGVSENNTPAMRRGPAGPRTLCNACGLMWANKGTLRDLSKGGRNLTMDHIEPGTPIEVKPLLVEGEFSGNQDEHGTLEGSSKTVIERSNDASVNLDEQDLHETAEDLTNSLPMGIVSSANDEQEPLVELTNPSDTDLDIPANFD*

>ChGATA02

MYGHSEPMTIPNPIPAGGDDDAAGPGVDSIDNAHIHYEPHTLEDGGGVVAVVEDVSSDPVYDVGSSEMRAQPYDGSSQLTLSFRGQVFVFDAVTPEKVQAVLLLLGGSELSSGPQGAELASQNQRGTEDFPIRCSQPHRAASLSRFRQKRKERCFDKKVRYSVRQEVALRMQRNKGQFSSSKKSDGDYSWGNGQESGQDDSHAETSCKHCGISSKSTPMMRRGPSGPRSLCNACGLFWANRGTLRELSKRTQDHSVTPAEQGEADTKDLNSVTAIDAHNSLVPFSNGDSSALVAEQ*

>ChGATA03

MEAPEYFQNSFCPQFPPEKRHSFDNNNNKATNGGGCGGDHFMVEDLLDFSNDDAVITDGGTTFDNVTGNSTDSSTLTVVDSCNSSSLSGSEPNVIPDIGSRNIAEGPFSSDLCVPYDDLAELEWLSNFVEESFSSEDLQKLQLISGMKARPDEAASETRQFQPEPNRNDNAHNTTTTNNNPIFNPDVSVPAKARSKRSRAAPCNWTSRLLLLSQPTSSSDQSDVVSSGPASPLQPPSTTGKKTVKSAPKKKESPEGPGGGPGDGRKCLHCATDKTPQWRTGPMGPKTLCNACGVRYKSGRLVPEYRPASSPTFVLTKHSNSHRKVLELRRQKEMVRAQQQFIHQVPPQQHHHHHHHHHQNMVFDVSNGGDYLIHQHVGPDFRQLI*

>ChGATA04

MMTPVYLNPASSPFSMVEQAEDQRLKLFISPPYDEAASCTSLPFPTFFDSLQDQTPGTTFTSLAHHQSQLYHHKDKNIWDCGTSYDQASSSSSLVQAHVVDAISNKDRRLSRCGDHERETNIGGEEEGKSSNYITRPRSVKWMSSKMRLMQKMTSNNPDLPPGTTDHIPAEISEHKFQIHAQPREISETSFSSNSNNTAAVRVCSDCHTNSTPLWRSGPLGPKSLCNACGIRQRKARRAMAEAAAAAANGFAVGSADTSSPRGKVAKEKKSRGSHKNKISKLIITDNASISHNNKKNNNNKKKICFKALDFQRVFPQDVAEAAMLLMELSCGLINNHS*

>ChGATA05

MLDPSDKGSESEEMNIKTPDVGSSEEGHKKTCADCGTSKTPLWRGGPAGPKSLCNACGIRSRKKRRAILGLNKENPNDKKGKRNKQLGDGLKQRLLALGREVLMQRSTVERQRRKLGEEEQAAVLLMALSYGSVYA*

>ChGATA06

MDPKGVQNGFEMTNFDQHEANIGGTDCLVDLTLRLGIPSSDKNNDQQSHTANGSSTSQAVDRSGLNNLNVNGYRQYEFPAPPELKNYCIINISNRRGKTGGSRKRKTTGRRPAKVGDIDRTCTNYNCRVTESPMWRTGPLGPKSLCNRCGIRFRKIKQKEETEQQAAVAAFKQLHAVSKL*

>ChGATA07

MTAVSADVTKLTKRENISAMASSMPTDDNFNIESFLPDELKALDFPMEDIEECVEDEDWYAQFQNLEPPSFEDLFCPRVLDGNDDMKPKNLSTSYGQTSQRNQLPRTAAKTTRGISSIRSEDLESIGSCAAQFEPKFEKRTRSKLSRSKRSSPAIFNTQFFPSTSSNSSASENLYNWDASESDLEGSLTEEMSNAAKRKQKKKRNLSQLSSAEMRKLSTEEPGESRETKRCMHCAVTKTPQWREGPLGPKTLCNACGVRYRSGRLFPEYRPAASPTFVASVHSNSHKKVIELRNKGCQGATMGILSSAQ*

>ChGATA08

MMNWGRRYVALDSVLGRKEPFPQAMNGNKKRCSDCMTTETPLWRGGPAGPKSLCNACGIRHRKRGIPTVSLMSKAPKRRREKTCGGSSSTITTTYNIGASATHNNASATTAKSTFGSGGGGINLNEPPKVRFVGYGEKVFLQDSQAEGEGEKQSQWREWGEVEQAAVCLLAMSCDSVFA*

>ChGATA09

MEDVYGGGASASSAQQQQDYFPIDDLLDLSNDDLFSSSTSSTDSIDLHPPPPPPHLHVSSTVFNPTAATDFTNDLCVPSDDVAELEWLSRFVDDSFTDFPTTNVFGSASFPNDTSSLFPSRVRTYRSKWGGPPEPSDSRAKPKREPSEASPSPSKPRRCAHCASEKTPQWRAGPMGPKTLCNACGVRFKSGRLVPEYRPAASPTFVLTQHSNSHRKVLELRRQKEASQQQQPEEQHQQKQQFYLHRDEYQVC*

>ChGATA10

MIGPNFIDEIDCGSFFDTIDDLLDFPNDDVESGLGPAPDCNAAFNNSLWPNQSGSLPAPNDAVFSAGNSASDLSAELSVPIEDIVQLEWLSNFVEDSFSGGSLTINKPDSFINKDTSHHQFRTSSPISVLDSSSSCSGDKNVPQSPGPVAAPGKRGRARSKRPRPATFNPRSAIQLISPASSVTEAEGPQAQPFLAPKAPSDSENFAESRPVIKIPKQASGEQKKKKKLKVSLPLAPLDGNQNSGPTQAAVRKCLHCEITKTPQWRAGPMGPKTLCNACGVRYKSGRLFPEYRPAASPTFVPSLHSNSHKKVLEMRTKGGELVVFGETATAMNETPELIPNTNSSISMDYM*

>ChGATA11

MQRCSSSSHGNMTGPCTCGVLHHTQSNSSFSMLFSMPNHHKPYDHHHHHHHETQHDHYNHMYPFASSSSSVDCTLSLGTPSTRLTENDVILDDKRTRNERRSVSNFCWDLLQPKHHATSATSSHHHKNGSHRSGGNSNGVSNAVHSNNDPLLARRCANCDTTSTPLWRNGPRGPKSLCNACGIRFKKEERRATAAAANGASSSVVGMEHNSHMLSQHHHNNSWMPHSQTQKMPCFSPAMSNEFRFMEDDTAHHENDATGIPFLSWRLNVTDRPSLVHDFTR*

>ChGATA12

MLYQTHHSFLFQFHPFTCSIPSSSSTTSTSTSTSPSPLSSLSPHPPLLSTRPPPLPPQQVGTEMECVEAALKTSIRKEMAVKASPQAVFDDLLWGGVNGQNGVACDDFSVDDLLDFSNEDGFVETEAEEDDKDKVKGFASVSPQKQPQDPENSDLSEKNELGPEPTSELSVPADDLENLEWLSHFVEDSFTEFTTSLPAGFIPEKPKTEKRPDPATPLPEKPCFKTPVPAKARSKRTRTGGRVWSLGSPSLTETSSSSSSSSSSSSPSSPWLIYATTQNREPAEAGGEPVGSVEKPPKKPKRRLVDGSSSQPPRRCSHCGVQKTPQWRTGPNGAKTLCNACGVRYKSGRLLPEYRPACSPTFSSELHSNHHRKVLEMRKKKDVTGVPEPGLTRPPVVPSFG*

>ChGATA13

MAPPSPFLELSGDGGDHDHQYHHLFNLEPQTSFSSSSLSSPLFLTPSQAQAPSDHYREPQNFQFQLLEADHHNIVSYGGSCDHDPQTLENESGSGTILKLSISKNGAGRNGNPRTDKWMSSKMRMMKKMTNPDKTSSSCTPSDDKPVAMKLSISHKSEEQKPQHPDMISCSNNSSNNMNNNVPIIRVCSDCNTTKTPLWRSGPRGPKSLCNACGIRQRKARRAMAAAAAAASGTTLAAAPSMKSTSKVQHKDNKPRVASTVPFKKRPYNKLSSTPPSKGRPPKKLCFEDFAISMNNNHSSSATTTTTTSLQRVFPQDEKEAAILLMALSCGLVHG*

>ChGATA14

MDDICGSDEPMHMNDNLHLQYMQEHEHHHGLDNISNVNGVADDHDNGSGGAELVQADVPSDPMNLSDTHDGMMDHGPENGDQLTLSFQGQVFVFDCVSPEKVQAVLLLLGGREVPPSMPAVPVTTQHANRGLTSTPQRLNVPQRLASLIRFREKRKERNFEKKIRYTVRKEVALRMQRKKGQFTSSKPNNDDSASAVTSSGSNESWSQDGNGSQHQEAVCRHCGINEKCTPMMRRGPDGPRTLCNACGLMWANKGTLRDISKAAAAAAAAPQAGQNPSISKNEDMKPDL*

>ChGATA15

MEMPEFYLGGYFDAGAAEFLPEKKPAAAAGDQFTVDDLLDFSNEDALVADGGFLDAAADSSAVTAVDSCNSSVSGGEPQFSGNRSFGDSQFSGDLCVPHDDLAELEWLSNFVEDSFSAEKDLQALQFLSMTTTKPQTPETSSSSETNQNAPLFHHETPLPGKARSKRSRAAPGDWSTRLLHLITPNDTIKPPKTTTCKKKDGIGGNSNSGSDSSGRKCLHCAAEKTPQWRTGPMGPKTLCNACGVRYKSGRLVPEYRPAASPTFVSARHSNSHRKVLELRRQKEVHRSHQHQFLSQSSIFGVSNGGHDEYLIHHHNVNDFRQMM*

>ChGATA16

MSESNHQNSMYGSGGAPQSNQVEEQEDDVEESIDNPHIRFEDSSAIPPNPLYLTSSEYPPAAVTNGGSDQLTLSFQGEVYVFDEVSPDKVQAVLLLLGGYEIPSGIPSMGPVPLNQQGMNDLPVKPIQPQRAASLSRFREKRKERCFDKKIRYTVRKEVALRMQRKKGQFTSSKASSDDGAPASSGATQGSGQDESMQETSCMHCGISSKSTPMMRRGPAGPRTLCNACGLKWANKGVLTGGPKVSNIGMQDPSAKGIEQGDGEAKDSVAITMGANIAPSSNGDNSAMTVDRNL*

>ChGATA17

MEMVNAQLLQARAYEGEDQLVHIPAVLEGDYDDGGGAKAAMNGGDQARRSSGVTMSRCNSALPSRTSELTIAFEGEVHVFPAVTPDKVQAVLLLLGRRDISSSFPSSESLLESNSGGIGDISRNSKLSRRTASLVRFREKRKERCFEKKIRYTCRKEVAQRMYRKNGQFASLKDDSKIASGNCDSSDGTSCPESVLRRCQHCGISEKSTPAMRRGPAGPRSLCNACGLMWANKGTLRDLTKPGRPIHFDQNELETAADFKPLMLKPENAHLDRDEEGSPEESKPIALDTENPPLRLGDEDMPETAEAAANHISIQMEDSTVNFDEQENLDEFCNASGTEFEIPANFDEQVVDFYDCNIETHWPGT*

>ChGATA18

MELCMEAKALKSSLRRELALKSNQQALIDEFWCATGISGVPSEDFSVDDLLDLSNGEFEDGSVEEEEEEEKDSVSVDDESSNSSNFVSTDSDSALASQLLVPDDDLAGLEWVSHFVDDSMLDLSLLHPVGTQKPEALALTPSETEAKPVQSRPTWFPSQVPVKPRTKRCRAASRVWSYPSSSSPSSSSSCSSGFSFSTPCLTFNNPVHSADVFVGEPATKKQKKKPAVQTGVDGSVGVQFQRRCSHCHVQKTPQWRTGPLGAKTLCNACGVRFKSGRLFPEYRPACSPTFSGDVHSNSHRKVLEMRKRKEAGGPEPGLNRVIPSF*

>ChGATA19

MDQRQKGSEAEHMNQSSIAPPSKSCTDCHTTTTPLWRGGPAGPRTLCNACGIKYRKRKRALVGLNKGATGTEKRKHKTIGAPLKLGLKSMEREMVEMVFHFHKKNLKEEEEAAILLMALSCSSGTGGAVYA*

>ChGATA20

MGKQGPCYHCGVTSTPLWRNGPPDKPVLCNACGSRWRTKGTLANYTPLHARAEPDDYEDHRVSRVKSISINKNKEIKLVKRKQNPDSVMVGGVAADYAHGFRKVTDEDTSNRSSSGSAVSNSESCAQFGSADASDLTGPAQSMVWDSMVPSRKRTCIGRPKPSPVEKLTKDLYTILHEQQSSYFSGSSEEDLLFECETPMVSVEIGHGSVLMRHPSSITREEESEASSLSVDNKQCHINEAYSHPETLLVHNNKGVIMTSTVTGKMNNLAGQGMQQEPLKRDKSQYDNFQIMGNHNSPLCHVDLNDILNFEEFTRQLTNEEQQQLLKHLPPVDVVKFPYSLKSMFDSPQFRENLTSFQQLLAEGVFDISFLGAKTEDCKTLKRLVLSNSSKSKWVERYHLLKKCKTSPGQSVISGPNALASSNFRHVKRLRDSETQNFPDVKIMMKSPKRIIVKGSNENKDLMDYDGSCFSPRSLFALPADGSSLLMESMNFVDESSDQDLLLDLPSNGSFAQAELLHPAMSFGAQQTSTSSSSIYPHVLHP*
